# Navigating the image discrepancy: A grounded theory approach to understanding Malaysia’s image among Chinese tourists

**DOI:** 10.1371/journal.pone.0324148

**Published:** 2025-05-27

**Authors:** Xiaocong Jiang, Ahmad Edwin bin Mohamed, Amirul Husni bin Affifudin

**Affiliations:** 1 School of Business, Suzhou Industrial Park Institute Vocational Technical, Suzhou, China; 2 School of Tourism, Hospitality and Event Management, Northern University of Malaysia, Sintok, Malaysia; Zhejiang Sci-Tech University, CHINA

## Abstract

The number of Chinese tourists visiting Malaysia has not returned to pre-pandemic levels, raising concerns that recent negative news stories widely circulated on Chinese social media may be a contributing factor. Due to the limited number of reviews, sentiment analysis struggles to determine whether negative news significantly affects tourists’ emotions and visitor numbers. This study employs empirical data to validate its impact, clarifying its actual influence on image discrepancy and tourist arrivals. Additionally, while managing image discrepancy is acknowledged as important in tourism, academic debate persists regarding whether negative news contributes to these discrepancies. This study conducted in-depth interviews with 30 participants, comprising 18 individual attendees and 12 attendees from 4 small seminars. Participants included recent visitors to Malaysia, prospective tourists who canceled their plans, and Malaysian tourism practitioners. Using the Context-Adaptive Grounded Theory (CAGT) methodology, the study explored whether sudden negative news events significantly influence Chinese tourists’ travel decisions and identified specific factors contributing to the discrepancy between Chinese tourists’ perceptions of Malaysia’s tourism image and the image projected by Destination Marketing Organizations (DMOs). The findings revealed that negative news was not the dominant factor driving image discrepancy. Instead, cultural differences—stemming from unclear understanding of religious and local cultures, as well as language barriers—were the primary causes. Additionally, inefficiencies in DMOs’ promotion of Malaysia’s multi-ethnic identity and an overemphasis on medical tourism, which failed to attract Chinese tourists, were identified as secondary factors. This study provides valuable insights into the complex impact of sudden events on tourism and offers guidance for Destination Management Organizations to better align projected and perceived destination images to facilitate tourism recovery.

## Introduction

Establishing and managing a tourism image boosts a destination’s competitiveness and directly fosters global tourism industry growth [1,2]. Post-pandemic, the tourism image’s role in destination recovery has grown critical, becoming a key driver in the resurgence of tourist arrivals and tourism economy recovery [3]. Tourism image construction is a multidimensional process involving both tourists’ perceived images and the images Destination Management Organizations (DMOs) intend to project. The interplay between these images is crucial in forming tourists’ overall impressions and the destination’s attractiveness [4,5]. However, discrepancies between perceived and projected images, often exacerbated post-pandemic, lead to what is known as image discrepancy [6,7]. These discrepancies usually negatively affect tourist arrivals [8]. Duan, Marafa [9] further suggest that negative emotions primarily cause image discrepancies, posing new strategic challenges in destination image construction and management.

China has emerged as one of the world’s largest outbound tourism markets, leading both in terms of travel frequency and expenditure [10]. According to the China Tourism Academy [11], the number of Chinese outbound tourists reached 87 million in 2023, recovering to 60% of the pre-pandemic levels of 2019. Approximately 93.95% of Chinese tourists prefer visiting neighboring countries or regions, with Malaysia being a popular destination [11]. Before the pandemic, China was Malaysia’s third-largest source of tourists, accounting for 11.9% of inbound visitors and contributing 17.8% to Malaysia’s tourism revenue [12]. In 2023, the proportion of Chinese tourists visiting Malaysia was only 30.1% of the figures from the same period in 2019. In contrast, the number of tourists from Singapore and Indonesia has seen a revival, reaching 58.07% and 61.06% of their pre-pandemic levels, respectively [13]. Therefore, effectively managing and aligning the consistency between perceived and projected images can not only provide Chinese tourists with a more satisfying and authentic travel experience but also bring long-term economic, social, and cultural benefits to the tourism destinations [5].

DMOs catering to Chinese tourists are facing new challenges in shaping tourism images. In 2023, the Chinese cinematic production “No More Bets” gained significant attention on Chinese-speaking social media platforms. The film explores the experiences of Chinese tourists on Malaysian islands, highlighting sensitive issues such as cyber fraud, human trafficking, and illegal organ trade. Additionally, reports of alleged exploitation by Malaysian immigration officials and inappropriate behavior of local tourism workers towards Chinese visitors have sparked considerable discussions on these platforms [14,15]. For instance, the 2017 Sabah boat sinking in Malaysia, which resulted in casualties among Chinese tourists, garnered significant attention on social media, rapidly heightening public concerns about travel safety [16]. Similarly, reports of a 2025 Kuala Lumpur street robbery also sparked heated discussions on Chinese social media, with hashtags such as “Malaysia is unsafe” trending, further highlighting the public’s heightened sensitivity to the safety of overseas travel destinations [17]. These incidents, combined with the film “No More Bets”, have contributed to Chinese tourists’ negative perceptions of Malaysia. Chen [18] and Lee [19] suggest that such news may impact tourist arrivals, but Kapuściński and Richards [20] argue that media sensationalism may amplify short-term risk perceptions, while its long-term effects remain unverified. Research by Chen [18] and Lee [19] has raised concerns within the academic community about the potential link between negative media coverage and the stagnant recovery of Chinese tourist arrivals. While the connection between negative reporting and image discrepancies has attracted widespread attention, its actual impact remains unclear. This study aims to empirically validate the actual impact of these factors in order to clarify their effects on image discrepancy and tourist arrivals. Therefore, investigating the factors affecting Malaysia’s tourism image discrepancy is crucial. Addressing these concerns is vital for adjusting strategies, optimizing tourist experiences, and promoting the recovery of tourist arrivals.

Nevertheless, current research on tourism destination image discrepancy exhibits several shortcomings. Firstly, existing literature suggests that questionnaire surveys are limited in revealing tourists’ deeper emotional responses [21]. Moreover, although previous studies have attempted to explore the link between tourists’ emotions and tourist arrivals using sentiment analysis of User-Generated Content (UGC) [22,23], the accuracy and effectiveness of these methods are questioned when comment data becomes scarce due to sudden events [24]. Consequently, there is currently no effective analytical method to ascertain whether the recent negative news event has elicited sufficient negative sentiment to prevent the recovery of Chinese tourist arrivals in Malaysia. Secondly, while studies have shown a positive correlation between negative news and tourists’ negative emotional responses [20,25], and negative emotions are identified as key factors causing tourism destination image discrepancy [5,26], there is yet no consensus in the academic world on whether negative news fundamentally affects tourism destination image discrepancy. Lastly, although research has extensively explored the issue of tourist arrivals in Malaysia from both the perspectives of perceived image [27,28] and projected image [29–31], there is a lack of in-depth analysis that simultaneously contrasts both dimensions and focuses on the angle of perceived versus projected tourism image discrepancy in examining the reasons behind the non-recovery of post-pandemic Chinese tourist arrivals to Malaysia.

We conducted in-depth interviews with Chinese tourists who recently visited Malaysia, those who planned to visit but were unable to do so, and local industry practitioners. The interview data were analyzed using an enhanced grounded theory methodology, exploring factors from the perspective of image discrepancy that affect the restoration of Chinese tourists’ visits to Malaysia and examining the potential link between negative news and destination image discrepancy. The grounded theory approach, especially valued for its ability to analyze complex phenomena affecting behavioral intentions in the context of limited data during sudden events, has gained widespread recognition [32,33]. This method not only uncovered the emotional responses of tourists but also provided an in-depth analysis of the core factors causing image discrepancy and affecting the recovery of tourist arrivals by identifying recurring themes and topics [33–35].

This study seeks to answer the following research questions:

Q1: Did sudden negative news events significantly impact Chinese tourists’ decision-making regarding travel to Malaysia, leading to a failure in the recovery of tourist arrival?

Q2: Can negative news events influence Chinese tourists’ perception of Malaysia’s tourism image, ultimately resulting in an image discrepancy?

Q3: What other specific factors contributed to the difference between Chinese tourists’ perception of Malaysia’s tourism image and the projected image by DMOs?

## Related work

### Image of tourism destination

The image of a tourism destination, defined as the comprehensive psychological impression held by tourists, encompasses multidimensional perceptions of the natural environment, cultural characteristics, and the quality of facilities and services [1,36,37]. Recent research has identified two core types, the perceived image formed by direct experiences and interactions, and the projected image disseminated by DMOs [4,5]. The interplay between these two images is crucial as they collectively determine the public’s overall perception and attractiveness of a tourism destination [7]. Identifying and assessing the consistency between the perceived and projected images is key to determining the success of destination image construction. However, existing research shows that there is often a significant discrepancy between the two images, leading to a mismatch between tourists’ expectations and actual experiences, which may result in disappointment or even aversion [4–6,9,38]. Additionally, the impact of the pandemic on the image of tourism destinations is significant. Studies suggest that the discrepancy between perceived and projected images may widen post-pandemic, posing new challenges for destination management [39,40].

In the study of Malaysia’s tourism image, diverse perspectives have been extensively examined, including those of local tourists [28], international tourists [41], and tourism policymakers [42]. Consistent findings highlight Malaysia’s rich cultural and natural resources, as well as its high-quality services, all of which have received widespread acclaim from visitors [28,31,41,43]. However, although DMOs are dedicated to promoting unique tourism attractions, including natural scenery [29], research indicates that tourists’ interests remain primarily focused on urban leisure tourism [43].

### Tourist emotions and tourist arrivals

Emotional responses experienced by tourists during their travels, known as tourist emotions, play a pivotal role in determining the quality of the tourism experience, satisfaction, and future behavioral intentions [44]. Recent research has highlighted the central role of emotions in tourists’ cognitive evaluations and behavioral responses, thus occupying a significant position in the study of tourist behavior [21]. Positive emotions, such as joy, excitement, and satisfaction, have been found to be closely associated with positive tourism experiences, increased destination loyalty, and enhanced propensity to recommend [21,45]. Conversely, negative emotions like fear, disappointment, and anger can lead to adverse evaluations of tourism destinations, reduced willingness to revisit, and the spread of negative word-of-mouth [25]. Particularly in the post-pandemic period, the impact of tourist emotions on tourism activities has become more complex due to uncertainties and health concerns brought about by the pandemic [46], highlighting the importance of understanding and focusing on tourist emotions.

Tourist arrivals typically refer to the number of visitors reaching a region, crucially reflecting the area’s attractiveness to tourists [27]. Post-pandemic, revitalizing tourist arrivals is considered a vital driving force for the economic and tourism industry recovery of a region [3]. Current research primarily focuses on forecasting trends in tourist arrival changes [3] and exploring strategies to enhance tourist arrivals [27]. Studies have identified various factors that can lead to a decline in tourist arrivals, including economic downturns at the destination, natural disasters, political instability, threats of terrorism, and health crises, especially public health events like the COVID-19 pandemic [3,27,47,48]. These findings not only provide a deeper understanding of the dynamics of tourist arrivals but also offer theoretical support for the development of effective strategies for tourism recovery.

The close relationship between tourist emotions and tourist arrivals has become a focal point of research for many scholars. Studies have revealed that positive emotions among tourists can significantly enhance the attractiveness of a destination, thereby increasing its tourist arrivals [49]. A possible explanation for this phenomenon is that tourists’ emotional experiences directly affect their loyalty to a destination; tourists with higher loyalty are more likely to make repeat visits or recommend the place to others, indirectly driving an increase in tourist arrivals [21,44,45]. Conversely, negative emotions can diminish a destination’s attractiveness, leading to a decline in tourist arrivals. This reduction is often due to tourists’ dissatisfaction or complaints, particularly when the travel experience fails to meet their expectations, thereby triggering negative emotions. Tourists carrying negative emotions are more inclined to share their adverse experiences, further impacting the destination’s image [23,50].

It is noteworthy that while both positive and negative emotions impact tourist arrivals, research indicates that negative emotions have a more significant and enduring effect on tourist behavior [25,49]. In recent years, some scholars [21,23] have explored the impact of tourist emotional expressions on social media, such as comments and discussions, on changes in tourist arrivals. Luo and Zhai [22] noted that UGC on social media, including comments and topic discussions, could amplify the impact of emotions on potential tourists, thereby further influencing tourist arrivals. Nonetheless, current research has yet to identify an effective method through emotional analysis for accurately pinpointing the specific causes of changes in tourist arrivals [51]. Part of the reason is that emotion analysis techniques struggle to precisely identify common themes and topics within negative emotions, limiting the ability to deeply investigate the specific factors triggering emotional changes [52,53].

Furthermore, the feedback on social media regarding sudden events often struggles to form quickly and adequately [54]. Especially for Chinese tourists, when evaluating tourism destinations, they tend to adopt more indirect feedback methods, even on social media platforms that offer anonymity. This behavior may be influenced by their collectivist and harmony-seeking culture, leading them to express emotions in a more subtle and conservative manner [45,55]. Therefore, when utilizing social media data to study whether sudden events have triggered negative emotions that significantly impact tourist arrivals, it may be challenging to obtain ideal research results.

### Negative emotions and image discrepancy

The academic community widely acknowledges that negative emotions significantly impact image discrepancy. Recent studies have underscored the central role of emotions in tourists’ cognitive evaluations and behavioral responses, significantly affecting tourist behavior [21,44]. Not only do tourists’ emotional experiences directly influence their perceptions and evaluations of a destination’s image, but image discrepancies can also, in turn, affect tourists’ emotions and experiences [5]. Although external factors such as environmental influences, inadequate marketing strategies by DMOs, and language and cultural differences affect image discrepancy [4,48,56], the subjective impact of tourist emotions on image discrepancy appears particularly significant [26].

The impact of negative news on tourist emotions is well-documented, with the style of presentation, tone, and dissemination method of news reports playing a significant role in shaping the public’s emotional responses [57]. Tourists’ emotional reactions are influenced by their personal experiences, emotional states, modes of information interpretation, and the content of the news [25]. Exaggerated or misleading negative reports can provoke disproportionate panic and anxiety, even when the actual risk is small, highlighting the importance of accurate reporting [58]. Research by Soroka and McAdams [59] suggests that people are more attentive to negative information, which implies that negative news is likely to elicit stronger emotional reactions [25]. Consequently, even exaggerated negative reporting can have profound effects on tourists, influencing their emotions and behavior. Media portrayal of dangers and crises can amplify perceived risks, thereby affecting tourists’ emotional responses [20].

Regarding the impact of negative news on the image discrepancy of tourism destinations, the academic community holds diverse and complex views. Some studies emphasize the significant impact of negative news on tourist emotions. For example, Fu and Timothy [60] propose that emotional reactions stimulated by negative news not only cause emotional distress but also play a decisive role in image discrepancy [44]. Chen [18] and Lee [19] further argue that the adverse emotional responses caused by these events damage the tourism image, leading to a decrease in the number of repeat visits by Chinese tourists to Malaysia. Zhang, Cho [61] extend this discussion by indicating that such emotional reactions can negatively affect Chinese tourists, potentially altering their perception and expectations of the destination.

However, other studies offer different perspectives. Tung, Tse [62] discovered that tourists might offset the impact of negative news through positive evaluations of other aspects of the destination, such as culture and environment, challenging the notion that negative news always leads to image discrepancies. Similarly, Xu, Lin [63] found a clear distinction between tourists’ safety perceptions and media-induced panic, suggesting that the impact of negative news on destination image discrepancy may be overestimated, providing a more nuanced understanding of the relationship between negative news and tourism image.

### Image of tourism destination and tourist arrivals

The relationship between the image of tourism destinations and tourist arrivals has been widely acknowledged by the academic community. The study by Huang and van der Veen [64] demonstrates that the image of a destination significantly influences tourists’ behavioral intentions, especially their intention to recommend, which directly impacts the destination’s tourist arrivals. Bigné, Sánchez [65] further confirmed that the tourism image serves as a critical antecedent for perceived quality, satisfaction, intention to revisit, and intention to recommend, thereby reiterating the crucial role of an excellent tourism image in boosting tourist arrivals. Additionally, Kamata [2] revealed the pivotal role of tourism image in the process of recovering tourist arrivals post-pandemic, highlighting the enduring importance of a positive destination image in attracting tourists even in challenging times.

Beerli and Martín [8] highlighted the relationship between destination image discrepancies and tourist arrivals. Sun, Tang [4] further clarified that these discrepancies could cause confusion among tourists, affecting their travel decisions. Therefore, understanding and managing destination image discrepancies is crucial for predicting tourist arrival fluctuations [5]. To increase tourist arrivals effectively, Sun, Tang [4] recommend that DMOs should implement strategies to minimize the gap between perceived and projected images and ensure the consistency and accuracy of marketing messages.

A series of measures aimed at shaping a friendly image of Malaysia have been empirically proven to effectively attract target tourist groups. These studies indicate that tourists highly identify with the social stability and rich natural tourism resources shaped by the country’s DMOs [27,29,30]. Furthermore, the quality of tourism services and experiences has also received high praise from visitors [28]. However, further research has revealed discrepancies between perceived and projected images, particularly in the portrayal of multicultural harmony by DMOs, which has not been fully perceived by tourists [29,31].

### Research gap

Although existing research provides valuable insights into Malaysia’s tourism image and tourist arrivals, several gaps remain.

First, the potential impact of sudden negative news events on tourists’ emotions and tourist arrivals has not been sufficiently validated. While existing studies primarily focus on macro factors, such as economic recessions and natural disasters, that affect tourist arrivals [3,27], there is a lack of systematic empirical analysis on whether sudden negative news significantly alters the travel decisions of Chinese tourists and obstructs the recovery of Malaysia’s tourist arrivals. Bushman and Pinto [66] suggest that the impact of negative news might be short-lived, and this opposing perspective warrants further exploration.

Second, the relationship between negative news and Chinese tourists’ perceptions of Malaysia’s tourism image, as well as the potential image discrepancy, remains unclear. Research has highlighted that social media platforms amplify negative emotions through user-generated content [20,21,58,67], potentially exacerbating the gap between perceived and projected images [5]. However, Tung, Tse [62] found that positive reviews can counteract such effects, and Xu, Lin [63] argued that there is a distinction between perceptions of safety and media-induced panic, suggesting that the impact may be overstated and requires further validation.

Lastly, the specific factors contributing to the discrepancy between Chinese tourists’ perceptions of Malaysia’s tourism image and the projected image by DMOs have not been systematically identified. While language barriers and cultural differences are often cited as key factors [4,48,56], Liu, Huang [23] noted that Malaysia’s religious and cultural appeal to Chinese tourists is limited, suggesting that the impact of cultural differences may be overstated, and that tourists’ preferences are more influenced by personal interests rather than a deep understanding of local culture. Similarly, Ferrer-Rosell and Marine-Roig [6] proposed that misconceptions about a destination are more often the result of tourists’ cognitive biases rather than deficiencies in the DMO’s projected image, emphasizing the dominant role of individual psychological factors in image perception. Moreover, Stylidis [68] found through empirical research that direct interaction with the local culture can positively shape tourists’ destination image, indicating that the role of DMOs communication may not be decisive, and that active tourist participation may have a greater impact. Jin, Wu [69] further questioned the significance of language barriers, suggesting that Chinese tourists’ language needs are not unique, and that in a globalized tourism environment, non-verbal communication may be sufficient to overcome communication obstacles. On the contrary, other scholars (e.g., Jiang and Mohamed [48], Jiang, Mohamed [70]) found that language communication barriers were one of the primary factors influencing Chinese tourists’ perception of tourism images in Malaysia. These opposing viewpoints suggest that the causes of image discrepancies may be multidimensional, and the contributions of external factors (such as language and culture) may vary depending on the tourist group and context. Scholars may have underestimated the dependence of specific cultural groups (e.g., Chinese tourists) on language support and cultural interpretation, indicating that deficiencies in DMOs communication remain a significant driver of image discrepancies in such contexts.

## Design

### The selection of methodology

In this study, we advocate for the application of grounded theory methodology to overcome the limitations inherent in current research approaches within the tourism image discrepancy domain. The choice of grounded theory is underpinned by three significant benefits. Firstly, the core of Grounded Theory lies in generating theories through theoretical saturation rather than the absolute scale of sample size [71], it facilitates the generation of precise research findings through the systematic examination of qualitative data, without necessitating extensive datasets. This quality makes it particularly relevant for investigating emergent phenomena not yet thoroughly understood [33]. Secondly, grounded theory is particularly well-suited for exploring complex social phenomena, such as tourists’ emotions and perceptions [33,71]. Lastly, it can help us to sift through the data for frequently occurring themes and topics, revealing the true causes of image discrepancy [35]. Grounded theory emerges as the optimal methodology for examining the intricate psychological responses of Chinese tourists to sudden adverse events.

### Technical adjustments to grounded theory

Although the grounded theory methodology holds significant advantages in research, we can still identify two potential improvements to enhance research efficiency and further increase its precision.

Despite the line-by-line coding required by grounded theory Glaser [33], it is found in actual coding that the content of each line of data is influenced by different text formatting. In many cases, the complete meaning cannot be expressed by only one line of data, and sometimes it requires multiple lines of data to extract a code. Therefore, Thus, inspired by Oliver [72], “progressive coding” is adopted in this study based on the principle of “not missing every valuable coding”, rather than copying the original coding method. For this reason, the coding was finished “line by line”, “sentence by sentence”, or even “word by word”.

Adjustments were also made to the recording and software. Glaser [33] did not recommend recording as a way for data collection, concerning that the interviewees might feel apprehension and could not express their true ideas. However, this study did not involve any personal privacy, and recording the interview can restore the original data to the utmost, which promotes the situation representation of the interview in the subsequent substantive coding and theoretical coding stages, contributing to the coding work. Non-disclosure Agreementswere signed before the interviews with respondents on the recordings, and the recordings would be immediately sent to them for confirmation to guarantee their legitimate rights and interests. The software of Nvivo 12 was introduced to convert the recording into text, which also made the coding more efficient. It should be noted that the coding of the collected text in this paper was completed manually instead of Nvivo 12. As Glaser [73] pointed out, cultivating theoretical sensitivity and extracting concepts from data to identify the relationships between these concepts and ultimately formulating a formal theoretical model is a capability that researchers must possess. It is also the foundation for the generation and transformation process of coding.

We adhere to the manual coding used in grounded theory but adopt a hierarchical coding methodology that utilizes recording technology, named Context-Adaptive Grounded Theory (CAGT). This adjustment further ensures that reliable theoretical insights can be generated through theoretical saturation, even with a relatively small sample size.

### Data collection

In this study, we adhered to the recommendations set forth by Baumer, Mimno [74], ensuring that participants recruited for interviews fully comprehended the research objectives. This approach also emphasized the diversity of participants and the richness of the information provided. To ensure theoretical saturation and the objectivity of sampling, we consulted the design suggestions regarding interview sampling, number, and duration from Thompson, Gage [75] and Seidel and Urquhart [76]. Throughout the sampling process, we continually compared data and guided the selection of subsequent interviewees based on the data already collected, ensuring each new participant contributed to the theoretical framework. This process persisted until theoretical saturation was achieved—point at which new data no longer significantly impacted the theoretical framework. This study was reviewed and approved by the Ethics Committee of the Suzhou Industrial Park Institute of Vocational Technology prior to commencement (Approval number: [sipivt202401025867]). During sampling, we rigorously adhered to the ethical guidelines of the Helsinki Declaration, safeguarding the privacy of all interviewees and ensuring their informed consent was obtained. This methodology not only supported the construction of theory underpinned by Grounded Theory but also ensured the research’s ethicality and effectiveness.

In this research, collaboration with the China International Travel Service Limited facilitated the identification of appropriate participants. Participant recruitment for this study began on January 2, 2024, and concluded on February 29, 2024. These individuals were categorized into two distinct groups for detailed interviews: Group A, representing the perceived image, and Group B, depicting the projected image. Group A consists of Chinese tourists who have visited Malaysia or planned to visit but did not, while Group B is composed of Malaysian tourism organizers with experience hosting Chinese tourists. These two groups represent perspectives on perceived and projected images, capturing key viewpoints related to Chinese tourists’ visits to Malaysia: Group A reflects the primary source of outbound Chinese tourists, while Group B embodies the intentions and practical experiences of destination management organizations. In the analysis, the perspectives of the two groups are integrated through the coding process. Group B’s professional experience complements Group A’s tourist perceptions, revealing the multidimensional causes of image discrepancy and ensuring a balanced representation of these differences. Interviews were conducted across Shanghai and Suzhou in China, and Kuala Lumpur and Penang in Malaysia. Inspired by Grosspietsch [77], Group A included tourists who visited Malaysia in 2023 and those who intended to visit but did not, maintaining an equal distribution between both subsets. This selection strategy aimed to diminish the influence of retrospective bias, ensuring that insights from both potential and actual tourists were comparably informative and contributed to a more nuanced understanding of the research theme. Moreover, this approach is in line with grounded theory’s emphasis on examining contemporary, context-specific experiences [75], facilitating a deeper exploration into the evolving perceptions of tourists.

Group A was first interviewed in Shanghai. As a city that has long been open to outbound tourism in China, Shanghai has accumulated many tourists with rich outbound tourism experience, which meets the basic requirements of purpose sampling. With this, Shanghai was our first choice to interview those who traveled to Malaysia and the interview content was studied as a sample. After the preliminary coding of the data, the relevant core categories emerge gradually, but a single sample is difficult to saturate and develop the categories. Therefore, we selected Suzhou as the second city to conduct interviews on the perceived image of Malaysia’s tourism. Similar to Shanghai, Suzhou also amasses people with abundant tourism experience as the first early cities to open to outbound tourism in China. The interference of other situational factors caused by different regions and environments can be avoided as these two cities are close physically and environmentally. The selection criteria for Group A focus on the perspective of Chinese tourists’ perceptions of Malaysia, which are directly related to the construction of Malaysia’s image. Tourists from Shanghai and Suzhou, due to their high level of engagement with Southeast Asian tourism, can provide rich data that supports theoretical saturation.

We designated Group B interviewees as Malaysian tour organizers with experience in hosting Chinese tourists. This approach aims to delve into these professionals’ understanding of the potential needs of Chinese tourists. It also facilitates an in-depth exploration of the intentions and strategies behind destination management, revealing the image of Malaysia they wish to present to the world. The selection criteria for Group B are directly based on the management perspective of Malaysia as a destination, focusing on the DMO’s projected intentions for the Chinese tourist market. This complements Group A’s perception-based perspective and collectively reveals the causes of the image discrepancy.

Interviews with Group B participants were initially conducted in Kuala Lumpur, Malaysia. Following a similar approach to Group A, the interview process continued in Penang, a location nearby, until data saturation was achieved. The choice of these two locations as survey cities for Group B was also because these two cities have a large number of tourism managers or practitioners who have accumulated rich experience in hosting Chinese tourists. Based on the research of Xu, Tian [78] that social media has a great impact on the new generation of Chinese tourists, this study also incorporated information posted by official or industry associations on social media platforms as part of the projection sources. The intentionally projected Chinese materials include Malaysia’s travel brochures, videos officially released by the Ministry of Tourism, Arts and Culture, Malaysia on the Chinese version of TikTok and Weibo (a Chinese social platform similar to Twitter), etc. The English materials are official tourism websites, publicity materials, and MTV for the four World Heritage Sites in Malaysia, etc. Materials with no intent for projection include a Chinese movie shot in Malaysia, a TV series that introduces early Chinese immigrants’ life in Malaysia, videos about the Malaysian local conditions and social customs by Chinese people in the Chinese version of TikTok, and travel notes and photos posted by Chinese tourists on Microblog. We submitted the sorted data to the respondents of Group B two weeks in advance for review. Additionally, the interview data from Group B provide insights into the DMO’s promotional strategies, such as the “Malaysia Healthcare” campaign promoted by the Malaysia Tourism Promotion Board on its official website and the Chinese version of TikTok.

### Data processing

**Fig 1:**
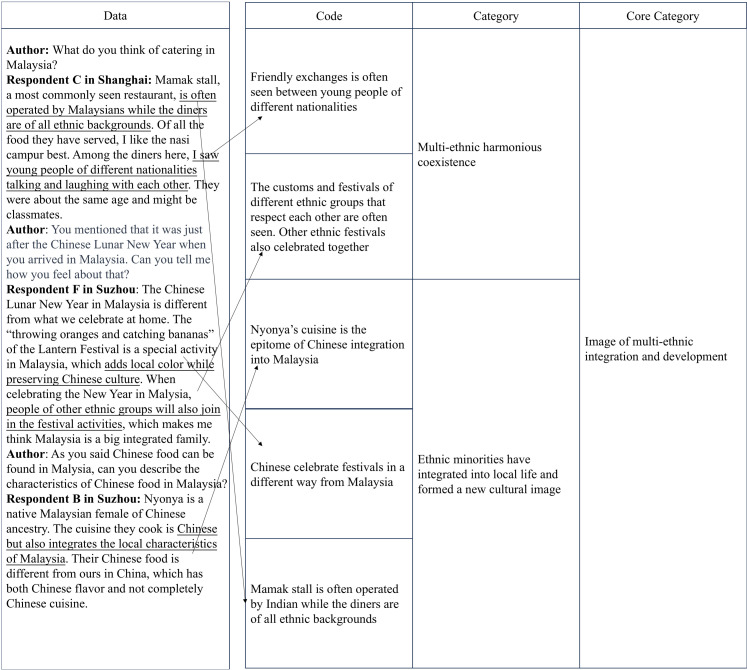
Example of coding process. Source: author’s own data analysis.

After the preliminary data collection, the data were processed and analyzed through substantive coding, which consisted of the stages of open coding and selective coding. The coding process was carried out independently by one of the authors, who was a native speaker of the language used in each of the two interview groups. In this way, the accuracy of the coding would not be affected [73]. This study was committed to the principle of “progressive coding” in the substantive coding stage, that is, the data was gradually abstracted and improved to make it more conceptualized. The example in Fig 1 demonstrated the process of discovering a core category “image of multi-ethnic integration and development”. The codes were extracted from the data of the interview and classified based on their characteristics and attributes in terms of their relevance. Those logically related codes were summarized, from which categories were extracted to discover the core categories through progressive induction. The data and codes in the figure are in line with the core category of “image of multi-ethnic integration and development”.

In the stage of open coding, this category was determined as the core category as it was supported by 40 codes or categories at seven levels. Then selective coding then focused on these core categories. At this stage, the core categories informed further data collection and theoretical sampling [73]. During the selective coding, the core category of the example in Fig 1 was considered saturated when it was supported by seven levels of 20 categories or codes. It was followed by the theoretical construction stage, in which the coding naturally emerged when extracting the critical path.

After obtaining the theoretical codes, the initially established theory was compared to the available literature to disclose and supplement the deficiencies in concepts, categories, and theories through a literature review. When new concepts and categories cannot be generated through continuous comparison, and the currently acquired data can be explained by the theoretical basis formed, the theoretical model reaches saturation, and the reliability of the theory also meets the requirements [75]. At this time, the theory construction was declared to be finished.

## Results

### Interview data collection and participants

As shown in Table 1, we interviewed 18 individual interviewees and 12 attendees in 4 small seminars, converting involved in our interview with a total of 280 thousand characters converted from the recordings. Small seminars captured group consensus on recent issues, while individual interviews provided insights into individual responses, revealing perception changes at the individual level. The combination of these two methods facilitates a comprehensive understanding of the potential impact of sudden negative events on discrepancies [79,80]. It is important to note that this study employs the CAGT approach, where data collection is not aimed at achieving complete parity in the number of interviews, duration, or transcription length between Group A (tourists) and Group B (practitioners). Instead, the study adheres to theoretical saturation as the criterion for ending data collection. As shown in Table 1, there are differences in the number of interviews, recording duration, and transcription length between Group A and Group B. This is because the focus of data collection is on the emergence and saturation of core categories, rather than the numerical balance between the two groups. In the analysis, the perspectives of the two groups are integrated through the coding process to reveal the dual causes of image discrepancy—perception and projection. This approach ensures the rigor of theoretical construction, rather than relying on the absolute equivalence of data quantity.

**Table 1:** An overview of interview.

Groups	Location	Number of Interviewees	Number of small seminars (number of attendees)	Total valid recordings (h/min)	Characters converted from recordings (10K)
Perceived image Group (Group A)	Shanghai	4	1 (3)	4h20’	16
	Suzhou	6	1 (3)	6h33’	
Projected image group (Group B)	Kuala Lumpur	3	1 (3)	3h22’	12
	Penang	5	1 (3)	4h17’	

### Results of the substantive coding stage

218 codes or categories at 7 levels were obtained in the open coding stage from the data of group A, followed by the selective coding stage after obtaining six core categories including “basic functional image”, “expectant service image”, “image of multi-ethnic integration and development”, “colorful tourism aesthetic image”, “language barriers affect image perception”, and “vague understanding of religious and local cultural image”. In the selective coding stage, these core categories were supported by 54 codes or categories and were determined to be saturated. For Group B, there were 207 codes or categories at six levels in the open coding stage. After obtaining six core categories, namely, “basic functional image”, “high-quality service image”, “tourism aesthetic image”, “new business forms of tourism image”, “damaged projection effect without Chinese”, and “vague image of religious and local tourism”, selective coding followed, in which these core categories were encouraged by 19 categories or concepts and were identified to be saturated.

**Fig 2:**
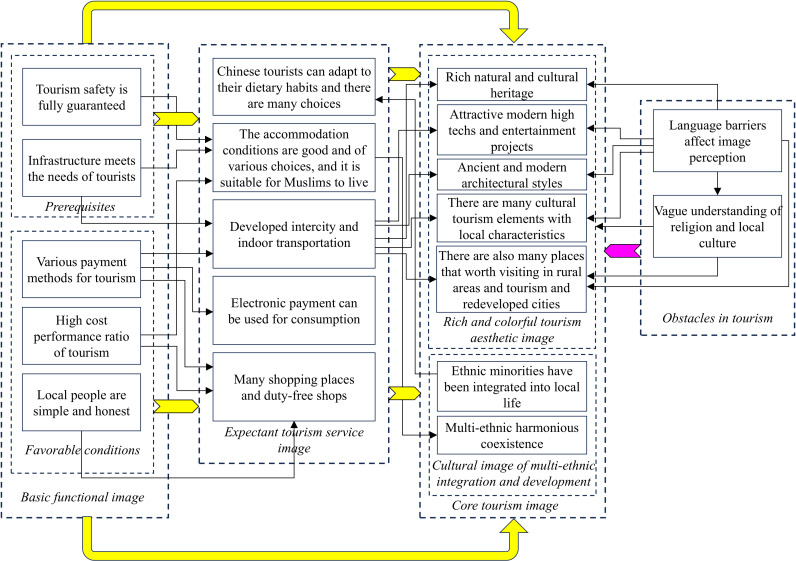
Schematic diagram of “tourists in focus” of projected image.

In Fig 2, the yellow arrow indicates that the basic functional and tourism service images can help tourists fully experience the core tourism image of Malaysia, and the purple one shows that language barriers and tourists’ insufficient understanding of religions and local cultures impede their recognition of the core tourism image. The process of obtaining the tourism image through tourists’ perception is termed “tourists in focus”.

**Fig 3:**
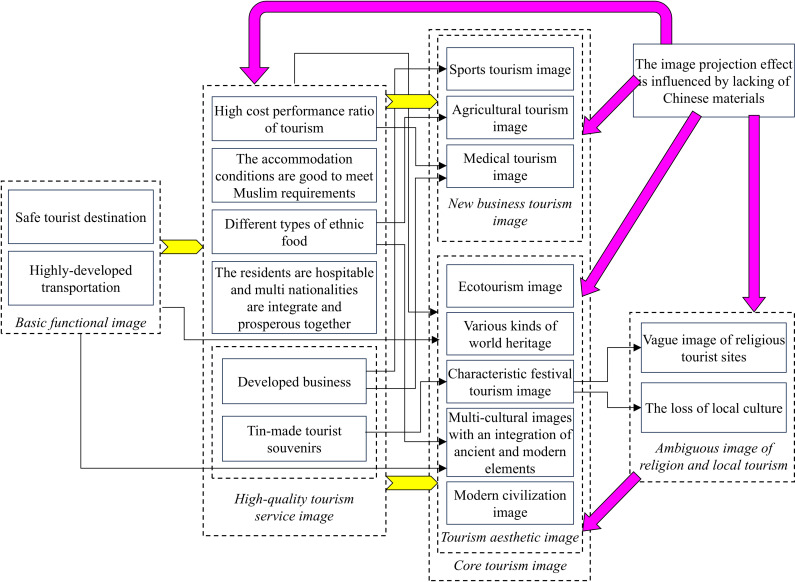
Schematic diagram of “host in focus” of projected image.

According to Fig 3, the new business type of tourism image and tourism aesthetic image is the projected core of Malaysia’s national tourism image. The basic functional image and high-quality tourism service image emphasize and guarantee the core tourism image, the positive impact of which is still indicated by yellow bold arrows. Moreover, the establishment of the core tourism image is damaged by factors of “The image projection effect influenced by lacking of Chinese-language materials” and “ambiguous image of religion and local tourism”. Language barriers are positively related to the tourists’ unclear understanding of religion and local images. The negative impact here is shown by the purple bold arrow. The process of obtaining the tourism image through destination projection is termed “hosts in focus”.

**Fig 4:**
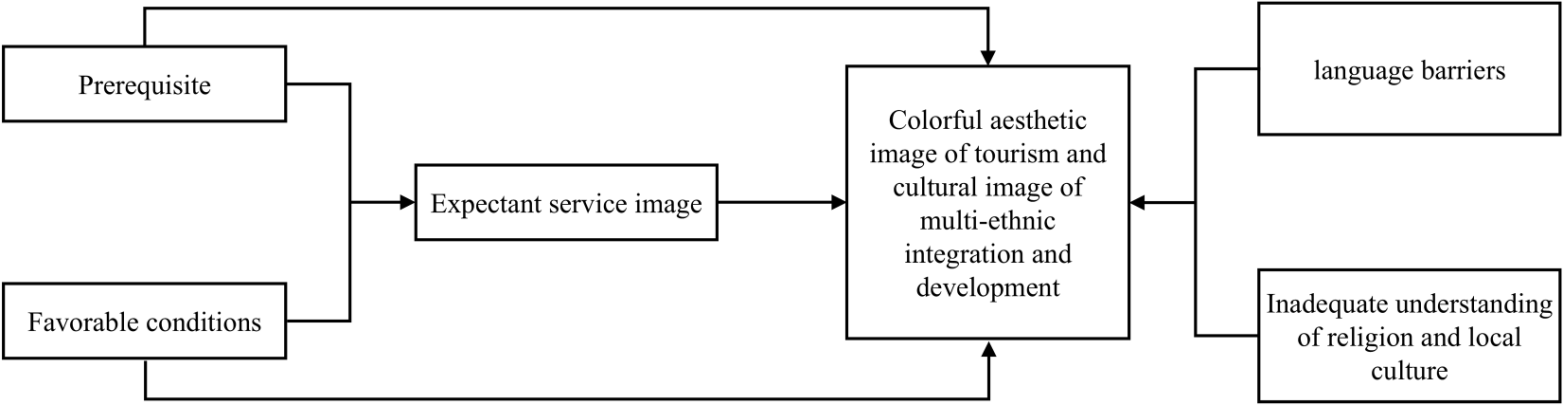
The evolution path of visitor in focus of image perception.

**Fig 5:**
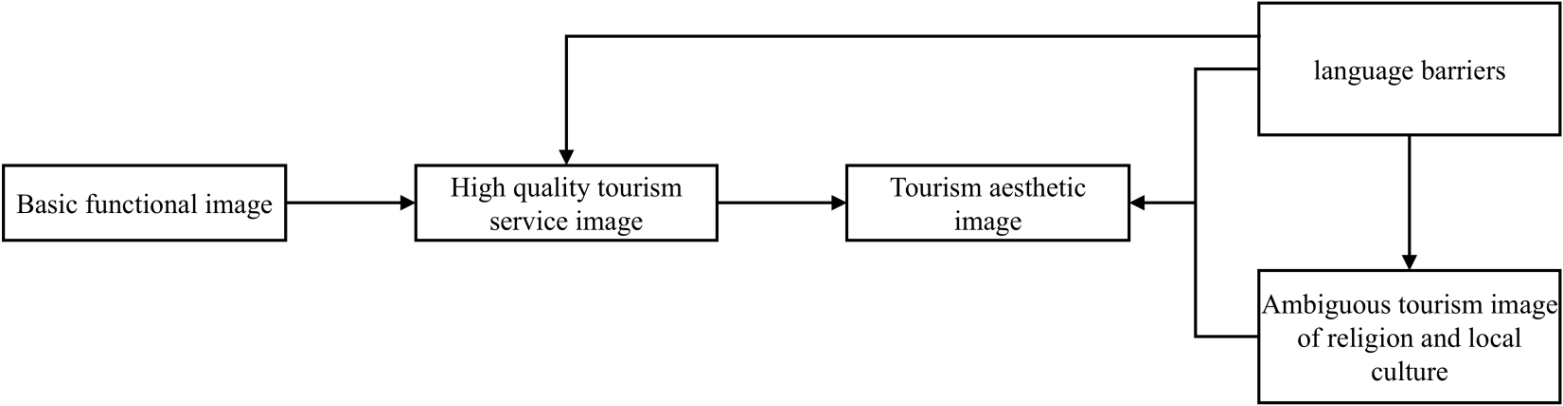
The evolution path of the host in focus of the projected image.

Following the design by Glaser and Strauss [71], we condensed the data during the final stage of substantive coding and extracted key pathways. By analyzing Figs 2 and 3, we clarified the logical relationships between the core categories, from which the evolution path of “tourists in focus” of image perception (Fig 4) and of “host in focus” of image projection (Fig 5) are obtained. As shown in Fig 4, both prerequisites and favorable conditions are the basis for displaying the image of tourism services and also support the rich and colorful tourism aesthetic image and the cultural image of multi-ethnic integration and development. However, language barriers in tourism and insufficient understanding of religion and local culture are also influencing the tourists’ in-depth understanding of the aesthetic image and cultural image of the country. The evolution path of the host in focus in Fig 5 is similar to that in Fig 4, so they won’t be covered here.

### Results of the theoretical coding stage

**Fig 6:**
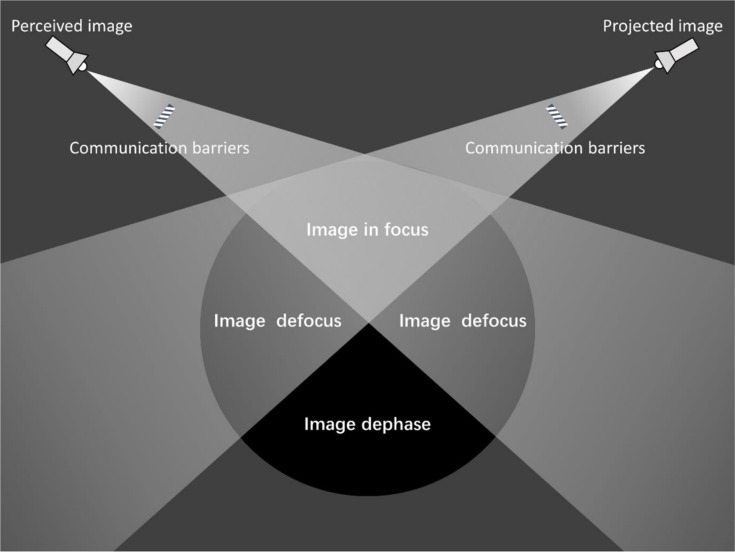
The model of tourism image with double focusing.

Drawing from the above two evolution paths, the codes and categories involved were sorted out to conduct theoretical coding. As for Group A, it was determined to be saturated as it was supported by 78 codes at 5 levels in the theoretical coding stage. Group B was encouraged by 118 codes at 6 levels and identified as saturated in the same stage. To ensure that theories naturally inducted from the data without the preconceived influence of existing theories, we undertook a literature review at this stage to prepare for constructing a theoretical model, a practice consistent with the design of Glaser and Strauss [71]. it found that the evolution path of tourists in focus was similar to the “tourist Gaze” obtained from the perspective of western tourists by Urry and Larsen [81] and of the host in focus resembled the “Host Gaze” obtained from the perspective of tourism residents in Moufakkir and Reisinger [82]. After rearranging and drafting the model diagram, a dual model of tourism image with a double focus was established and named as the model of tourism image with double focusing (Fig 6).

Groups believe that Malaysia boasts a full and varied tourism “aesthetic image” and “good service” image. where the image experienced by respondents in Group B is highly recognized by those in Group A. Second, little attention from interviewees in Group B has been paid to the image of “multi-ethnic image of integration and development” that is perceived by respondents in Group A, and the “new business tourism image” in the projected image has not been perceived by respondents in Group A. Third, both groups agree that discrepancies in the image of religion and local culture exist, with “poor understanding of religious culture” and “blurring of indigenous culture” identified as the two main reasons for this situation. The issue of “blurring of indigenous culture” primarily focuses on tourists’ “unfamiliarity with the daily life situation of rural and non-tourist city dwellers” and the “lack of presentation of local cultural tourism resources.” Furthermore, both groups of respondents unanimously believe that “language barriers” limit Chinese tourists’ perception and understanding of the projected image of the tourism destination. The interview data clearly reveal how language barriers and cultural differences impact Chinese tourists’ perceptions of Malaysia. For example, one Group A respondent described, “I wanted to buy something at a market in Penang, but the vendors only spoke Malay. I tried gesturing for a long time, but couldn’t understand the price, so I had to walk away.” Another Group A respondent mentioned, “I really wanted to understand the significance of local festivals, like Eid, but there were no Chinese-speaking guides at the attractions, and I was completely lost.” These examples illustrate how language barriers limit tourists’ basic interactions and information access. Regarding cultural differences, one respondent from Group A said, “I saw many people wearing traditional clothing, but no one told me what these garments meant. I felt like I missed out on a lot.” Another noted, “I thought Malaysia had a very serious religious atmosphere, but after interacting with the locals, I realized they were very warm and welcoming. It’s a shame no one explained this to me beforehand.” Tourism professionals from Group B also corroborated this phenomenon. One respondent remarked, “We rarely introduce the cultural backgrounds of mosques or traditional villages to Chinese tourists, so they naturally find these places unfamiliar.” These interview records collectively support the study’s conclusion that language barriers and insufficient understanding of religion and local culture are key drivers of image discrepancies.

Furthermore, the disappointment or fear caused by language barriers and cultural differences exacerbates the perception and projected image discrepancy by reducing engagement and willingness to explore. One respondent from Group A stated, “We heard that Malaysia has rich traditional festivals and folk activities, but when we arrived, we didn’t see any related events. We were disappointed and didn’t want to stay any longer.” This disappointment, caused by a lack of cultural experiences, reduced their desire to stay. Another respondent mentioned, “We got lost in Penang, wanted to ask for directions, but no one understood Chinese. We didn’t know what to do and felt anxious, eventually giving up on exploring.” This fear, triggered by language barriers, restricted their freedom of movement. Group B practitioners also confirmed this, with one respondent noting, “Some Chinese tourists, because they cannot understand the explanations or find Chinese information, seem very uneasy and quickly lose interest in the attractions.”

Conversely, the research findings indicate that negative emotional reactions triggered by negative news are not the main reasons affecting the discrepancy between perceived and projected tourism images. The interview data reveal how specific emotions, such as disappointment and fear, influence the behavior of Chinese tourists in certain contexts. For example, one respondent from Group A stated, “I heard that some areas in Malaysia have safety concerns, and I felt a bit scared, so I didn’t dare visit rural attractions and stayed in the city instead.” Another Group A respondent mentioned, “I was really looking forward to experiencing the local culture, but when I arrived, I found no one could explain anything, and even basic communication was difficult. I felt disappointed and lost interest in learning more.” Tourism professionals from Group B also observed similar phenomena. One respondent noted, “Some Chinese tourists, after arriving at the attractions and finding they couldn’t understand the guides, became disappointed and lost interest in our activities.”

### Validation of the results

In accordance with Venkatraman and Ramanujam [83], we validated our findings by contrasting the discrepancies between perceived and projected images with outcomes from a singular dimension discussed in prior studies. As illustrated in Table 2, although an exact match with previous research is not always present, our results align with multiple earlier works. Specifically, the concordance between perceived and projected images in our study echoes the findings of ref. Ghani [27–30]. Additionally, our insights on the “multi-ethnic image of integration and development” alongside the “unfamiliarity with the daily lives of rural and non-touristic city residents” are in agreement with the research conducted by Hussin [29] and Hassan [31]. Therefore, the CAGT method’s results are corroborated by these studies.

**Table 2:** Comparison of results obtained through CAGT by analyzing image discrepancies with those obtained from a single perspective.

This study (by GT)	Ghani [27] Perception of Tourism Image	Liat, Mansori [28] Perception of Tourism Image	Hussin [29] Projection of Tourism Image	Zainuddin, Saad [30] Projection of Tourism Image	Hassan [31] Projection of Tourism Image
1.1. Colourful aesthetic images	Beautiful leisure and environmental attractions		Cultural and natural tourism resources are important tourist attractions	Natural beauty, proximity to nature, and weather, etc	
1.2. Good service image	Good holiday tourism destinations, high-quality tourism services	International tourists are satisfied with the travel experience		Destinations with high-quality tourism experiences	
2.1. New business tourism image	N/A	N/A	N/A	N/A	N/A
2.2. A multi-ethnic image of integration and development			Image of multi-ethnic integration and development		DMO projecting an image of multi-ethnic integration and development to potential tourists
3.1. Poor understanding of religious culture	N/A	N/A	N/A	N/A	N/A
3.2.1. unfamiliarity with the daily life situation of rural and non-tourist city dwellers	N/A	N/A	DMO committed to promoting unique tourist attractions including daily life in rural and non-tourist cities	N/A	N/A
3.2.2. Lack of presentation of local cultural tourism resources	N/A	N/A	N/A	N/A	N/A
3.3. Language barriers	N/A	N/A	N/A	N/A	N/A

Our research findings demonstrate considerable comprehensiveness, largely attributable to two key factors, the application of the CAGT methodology, which provided us with profound insights and understanding. Additionally, an in-depth understanding of image discrepancy has revealed how strengthening marketing strategies can effectively increase destination tourist arrivals [4]. Consequently, our study uniquely identifies four image discrepancies, “New business tourism image,” “Poor understanding of religious culture,” “Lack of presentation of local cultural tourism resources,” and “Language barriers”, as the primary reasons for the failure to recover tourist arrivals.

## Discussion and implication

The influence of negative media coverage on destination image has been widely discussed, yet its specific effects are often shaped by various factors, such as context, content, and timing [8]. Fairley, Lovegrove [84] highlight that negative coverage can swiftly evoke emotional responses among tourists, particularly when the coverage spreads rapidly or pertains to a severe incident. Under such conditions, a short-term impact on destination image is common. However, over time, especially with crisis management interventions, the influence of negative news often diminishes or even dissipates [24].

Our findings further align with this perspective. We observed that, while initial negative coverage indeed triggered brief concerns among some Chinese tourists—especially as negative content spread quickly on social media, sparking a certain level of doubt regarding the destination—these concerns did not ultimately lead to a long-term deterioration in Malaysia’s image. Blackman, Kennedy [24] noted that the impact of negative news diminishes over time and with effective crisis management. Our interview data support this perspective. For instance, one Group A respondent stated, “I was a bit scared when I saw the news about the shipwreck, but after doing some research, I felt better,” indicating that negative news only triggers short-term concerns and does not have a significant long-term impact on the image. Another respondent mentioned, “The security issues seemed serious, but I don’t really believe it without personal experience.” This aligns with the views of Kapuściński and Richards [20], who argued that media exaggeration may amplify short-term risk perceptions, but its effects are often intensified by cultural misunderstandings and other factors.

This phenomenon suggests that the impact of negative media reports may not be as enduring as initial reactions might indicate; rather, it is moderated and mitigated by a range of factors. These results also echo the findings of Muhoho-Minni and Lubbe [85], affirming that the transient nature of such media impacts may be a generalizable pattern in destination image resilience. Therefore, in addressing image discrepancy, DMOs should prioritize resolving deeper structural issues rather than focusing excessively on the short-term fluctuations caused by negative news. This study clarifies that negative news is not the primary driver of image discrepancy; its impact is more transient and contextual, rather than decisive.

Our results reveal that Malaysia’s rich natural and cultural tourism resources are not only accurately presented by DMOs but also the perceived by tourists is fully aligned with this presentation, a phenomenon we term as “image focus”.

In discussing the impact of media reporting on destination image, Moyle, Moyle [44] highlighted the significant negative effects of bad news, while Zhang, Cho [61] further revealed how such reporting adversely affects the tourism image, leading to decreased interest among Chinese tourists in the destination. However, our study offers a different perspective. Our findings suggest that the aforementioned factors are not the main reasons behind destination image discrepancy and the subsequent failure to recover tourist arrivals. Thus, our research suggests that DMOs should not overly focus on the impact of negative news on tourist arrivals, providing a new viewpoint for destination management and recovery strategies in the tourism industry.

### Discussion of key findings

#### The phenomenon of image dephasing as the primary cause of image discrepancy.

In our research, we introduce the concept of “image dephase” to capture the disconnect between the perceived and projected images of a destination. Fig 6 reveals that tourists, despite their interest, often fail to fully engage with the destination’s cultural essence due to a limited understanding of its unique culture. Furthermore, we highlight the inefficiencies of DMOs in accurately promoting the unique cultural aspects and appeal of destinations, which hampers the effective communication of their authentic charm to tourists. Our analysis identifies that negative news is not the primary driver of image discrepancy, language barriers and cultural differences as the main contributors to this phenomenon.

This research examines the detrimental effects of language barriers as communicative impediments in forming the perception of tourist destinations. Contrary to Jin, Wu [69], who suggest the linguistic needs of Chinese tourists are not distinctly unique, our findings indicate that language barriers considerably hinder Chinese tourists’ comprehensive understanding of destination images. This observation is in line with Ying, Wen [56], who found similar impacts among Chinese tourists in New Zealand, thus underscoring the significant role of language barriers in shaping destination images. According to the results, the interviewees in Group A believe that the lack of Chinese introduction affects tourists’ perception of tourism image, For example, one respondent from Group A recalled, “I saw many interesting foods at the night market in Kuala Lumpur, but there were no Chinese menus. I didn’t dare to order randomly and ended up just having the familiar fried rice.” These experiences suggest that language barriers cause tourists’ perceptions to remain superficial, leading to a discrepancy with the rich image that DMOs aim to convey. This finding aligns with the research by Jiang, Mohamed [70], which identified language barriers as a major challenge faced by Chinese tourists in Malaysia. Due to insufficient language support (e.g., lack of Chinese signage in shopping areas and unclear categorization of halal restaurants), Chinese tourists develop misunderstandings of Malaysian culture, significantly affecting their sense of cultural connection to the destination. Those in Group B also consider the lack of Chinese projection materials influences the projection effect, which demonstrates that most Chinese tourists are not equipped with sufficient English skills to communicate effectively in traveling. A typical statement is as follows: “I originally wanted a blanket, but I couldn’t speak, so I gestured for a long time and the waiter brought me a towel.” Another example is: “I wanted to try the local cuisine, but many times their restaurants don’t have menus in Chinese, and I can’t understand the English spoken by the waiters either.” Accordingly, this result is also consistent with the survey of Chinese tourists conducted by Jiang and Mohamed [48] in Malaysia.

Cultural differences represent another factor contributing to image discrepancy, one respondent stated, “I was really curious about Malaysia’s multiculturalism, but the guide only took us to modern shopping areas, and we had no opportunity to experience authentic local life.” These experiences suggest that the lack of cultural engagement leads to superficial perceptions, resulting in a discrepancy with the rich image that DMOs aim to convey. aligning with the perspective put forth by Sun, Tang [4]. The study findings reveal that cultural differences lead to a poor understanding of religious culture and the blurring of indigenous cultures, restricting Chinese tourists’ deep understanding and appreciation of Malaysian culture. Chinese tourists express interest in Islamic architecture and customs but fail to deeply understand their significance due to a lack of knowledge about Islamic doctrines. For example, one respondent stated, “I really appreciate the design of the mosque, but I know nothing about its cultural meaning.” This aligns with the view of Koufodontis and Gaki [86], who argue that cultural differences are the root cause of understanding barriers., the results indicate that while Chinese tourists show interest in Islamic architecture and Muslim customs, they often fail to grasp the cultural significance during their travels due to a lack of understanding of Islamic teachings. This perspective is supported by the research of Koufodontis and Gaki [86], which identifies cultural differences as the fundamental cause of this comprehension barrier. Liu, Huang [23] believed that local religious culture cannot appeal to Chinese tourists. Still, in this study, the results show that Chinese interviewees take to local religious culture whether they are Muslim or not. Here is a good example: “I admire their mosques, both in terms of architectural design and Islamic teachings. I believe that the local residents’ devout and gentle nature might also be influenced by their religion.” Another example statement is: “I love the design of mosques, but I have no knowledge about their culture.” This fact highlights the importance of establishing an all-round religious and cultural image in the process of promoting Malaysia’s national tourism image. Nevertheless, Islamic culture is not projected to Chinese tourists as part of Malaysia’s national tourism image, but only projected to Muslim countries in the Middle East and North Africa [27].

This study finds that Chinese tourists are greatly interested in the indigenous cultural characteristics and lifestyles of the local people. A typical statement is:“The travel agency doesn’t offer any tourism activities that allow experiencing the living conditions of the local Malay people, but it’s enticing.” This finding not only reveals tourists’ significant preference for non-sightseeing tourism experiences but also resonates with the findings of Stylidis [68], which indicate that tourists’ in-depth experiences with a destination’s local culture can positively influence their perceptions of the destination’s image. A practitioner from Group B also mentioned, “The tour routes we design rarely include religious activities or traditional craft displays, as we assume Chinese tourists may not be interested. However, this actually deepens their misunderstandings.” These findings are highly consistent with existing research. An and Lin Tan [87] found that when a destination fails to communicate and provide experiences related to local culture, Chinese tourists’ satisfaction and interest decline. Additionally, Li and Kwortnik [88] emphasized that the complexity of religious culture, if not properly explained, can lead Chinese tourists to view it as unfamiliar rather than appealing. These studies support the conclusion of this research, which suggests that the lack of language support and cultural communication by DMOs directly contributes to image dephase. Our study further substantiates this point, emphasizing the potential role of Malaysia’s DMO in designing tourism products that deeply engage with local culture. However, despite significant market interest, we find that Malaysia’s tourism industry managers still lack in seizing this opportunity and providing services that cater to this emerging demand. This service deficiency not only limits tourists’ potential for in-depth exploration of Malaysian culture but also affects their willingness to revisit or recommend the destination to others, thereby impacting the recovery of tourist arrivals.

It’s also discovered that the DMOs in Malaysia never construct an exotic cultural image and accordingly project it to Chinese tourists, which dovetails with the findings in a study concerning Indian tourists by Kiran Sarkar Sudipta, Kumar Lenka Sarat [89] that Malaysia’s national tourism image is short of tourist resources about local culture. This study further reveals that Malaysia’s destination may suffer negative impacts on its overall tourism image due to its inadequate showcasing of its indigenous cultural features, particularly concerning Chinese tourists seeking deeper cultural experiences. This finding aligns with the results of Chen and Rahman [90], who found that when a tourist destination fails to adequately showcase its unique indigenous cultural characteristics and way of life, it may lead to potential tourists turning to other places better suited to satisfy their cultural exploration needs.

Furthermore, this study reveals that the image dephase phenomenon is not only a result of insufficient information transmission but also significantly affects Chinese tourists’ decision-making behavior by triggering specific emotions such as disappointment and fear. Language barriers and the lack of cultural information are the primary triggers of disappointment. Li, Scott [21] argue that disappointment, as a core emotion in the tourism experience, not only directly weakens immediate satisfaction but also has a profound impact on tourists’ cognitive processes and behavioral decisions through a mediating effect. In this study, this effect is evident as Group A respondents shorten their stay due to the lack of promotional festival activities, demonstrating how disappointment extends from the perceptual level to the behavioral level. Similarly, Nawijn and Biran [25] further explain the impact mechanism of fear in their research on negative emotions. They found that fear (e.g., anxiety from getting lost due to language barriers) activates the psychological avoidance mechanism, weakening tourists’ emotional attachment to the destination and prompting them to favor destinations perceived as lower in risk. In this study, fear not only limited immediate exploratory behaviors (e.g., abandoning unplanned attractions) but may also reduce Chinese tourists’ intention to revisit the destination through long-term emotional memories. As one respondent stated, “Next time, I would rather go to Thailand; at least language communication isn’t such a big problem.” These findings align with those of Li, Hernández Martín [91], who observed that negative emotions like disappointment and fear significantly reduced Chinese tourists’ trust in and interest toward the destination. Zamor, Nicholls [92] further add that when cultural misunderstandings or communication barriers trigger negative emotions, tourists tend to reduce their investment in the destination and may even opt for more familiar alternatives. Observations from Group B practitioners also support this view, as they noted that tourists lose interest when they cannot understand the guides, which weakens their identification with the image projected by the DMO. In conclusion, if DMOs fail to effectively address disappointment and fear, it will not only exacerbate image dephase but may also lead to lasting negative effects on tourists’ long-term decision-making.

The phenomenon of image defocus as a secondary factor in generating image discrepancy. In the construction of Malaysia’s tourism image, two inefficient scenarios exist. Firstly, tourists may perceive a certain image, which may not necessarily be the image deliberately promoted by the DMO. Secondly, certain images intentionally promoted by the DMO may not be accurately perceived by tourists. For instance, “the multi-ethnic image of integration and development” and the “new business tourism image” exemplify this phenomenon. We term such scenarios “image defocus,” as illustrated in Fig 6, which reveals the information transmission gap between perceived and projected images, in which the relationship between image projection and perception has been one-sided. This situation is typically characterized by a lack or low efficiency of image projection.

Previous studies have shown that a relatively stable social environment is positively correlated to national tourism image [41], and this study proves that ethnic integration promotes social stability which further contributes to sound national tourism image. A typical discussion contains the following: “In Malaysia’s Chinese cuisine, there are unique dishes that incorporate Malay and Indian cultural elements, such as Nyonya dishes and Malay-style fried noodles.” These dishes showcase the diversity and inclusivity of Malaysian society, providing visitors with a rich culinary experience while conveying the message of integration and harmony in Malaysian society. Another example is: “During the celebration of the Chinese New Year in Malaysia, people from other ethnicities also participate in the festive activities.” This study discovers that the harmonious coexistence of the three ethnic groups in Malaysia mirrors a fair trend of social integration and development, and ethnic minorities and the large population of Muslims can live in harmony. This ethnic integration and collective participation strengthen social cohesion and stability, while also showcasing to visitors an image of a nation where diverse cultures coexist harmoniously. Previous studies revealed that Islamic countries are faced with restrictions on tourism development considering the specific teachings of Islam [93], and the gap between ethnic minorities and Muslims hinders the building of a national tourism image [94]. On the contrary, this study finds out that there is no gap between Malaysian people, and ethnic minorities have fully integrated into Malaysian society and secured new development, so that Malaysian tourism image is positive on the whole, which however, escapes the notice of respondents in Group B. That indicates that the work efficiency of the DMO in Malaysia needs to be improved. One the other hand, the DMO in Malaysia are sparing no effort to introduce to Chinese tourists new forms of tourism. Previous studies have considered such new forms of tourism as medical tourism will become the highlights attracting Chinese tourists after the pandemic of COVID-19 and also positively help reshape Malaysia’s national tourism image [95]. However, the interviewees in Group A never participated in or heard about tourism of the new forms like agricultural, medical, or sports tourism when traveling in Malaysia. For example, the Malaysia Healthcare Travel Council (MHTC) has been actively promoting medical tourism in recent years by partnering with the Chinese market to showcase Malaysia’s cost-effective medical services and advanced facilities. In collaboration with the Malaysia Tourism Promotion Board, MHTC has conducted a series of promotional activities in several cities in China, such as Guangzhou and Shanghai, aiming to enhance Chinese tourists’ awareness and trust in Malaysia’s medical tourism offerings [96]. However, one respondent from Group A stated, “I’ve only seen medical tourism ads online, but no one recommended it during my trip, and I didn’t know where to go for such experiences, so I ended up visiting Sabah for its natural scenery.” Another respondent mentioned, “I was more interested in local night markets and traditional festivals. Medical tourism did not appeal to me.” This suggests that the DMO’s efforts to promote a new tourism image have not effectively reached Chinese tourists. Instead, these promotions are disconnected from the tourists’ actual interests in cultural and natural experiences, further exacerbating the image discrepancy between perception and projection. The above situation accords with the conclusion drawn by Peng, Yang [97] that the domestic medical tourism system in China is being established and its popularity among the public is on the increase year by year. Nowadays, the medical tourism systems in South Korea, Thailand, and Taiwan are more popular with Chinese tourists than the medical tourism in Malaysia [98,99]. The above demonstration manifests that a majority of Chinese tourists have no interest in the tourism of new forms, which, therefore, fails to effectively attract Chinese tourists to Malaysia at this stage.

### Theoretical contribution & practical implication

Our work contributes to the literature in the following ways.

Firstly, this study, through the use of the CAGT methodology, reveals the significant impact of cultural misunderstandings and language barriers on the discrepancy between Chinese tourists’ perceived image and the image projected by DMOs, thereby extending the application of image discrepancy theory in the context of cross-cultural tourism. This methodology overcomes the limitations of sentiment analysis when comment data becomes sparse due to unforeseen events, thus enhancing the understanding of how such events affect fluctuations in tourist arrivals.

Second, we enrich the discourse on destination image discrepancies by examining the origins of negative emotions. Prior studies have mainly focused on how negative news affects tourist emotions [8,21,77] and the role of these emotions in image discrepancies [20,25]. The academic debate continues over whether emotional responses to negative news are a significant factor in creating image discrepancies. Our research indicates that it is not the primary cause. This clarifies that while negative news may affect tourists’ perceptions to some extent, it does not ultimately lead to a significant image discrepancy, thereby refining the nuanced relationship between emotions and image discrepancy. Thereby extending the investigation into the relationship between tourist emotions and image discrepancies.

While previous studies have examined the relationship between Malaysia’s tourism image and tourist arrivals, they primarily focused on either perceived image or projected image as independent perspectives [27–31]. In contrast, our study delves into the discrepancies between perceived and projected images, specifically by analyzing the factors underlying these differences to identify the reasons why tourist arrivals from China to Malaysia have not rebounded. We identify additional factors such as cultural misunderstandings, language barriers, and emotional responses like disappointment and fear as key contributors to this discrepancy, offering a multi-dimensional explanation for the persistent decline in tourist arrivals.

Based on our findings, we advise DMOs not to overly concern themselves with the potential threat of negative news on social media. Our research identifies the key factors affecting the lack of recovery in tourist arrivals to Malaysia, thereby offering practical insights and recommendations for DMOs, including those in Malaysia, with China as a primary source market:

1. Meeting language needs: Considering the limited English proficiency of Chinese tourists, tourism management departments in destinations can take measures to improve the experience for Chinese tourists and reduce the image discrepancy caused by language barriers. These measures may include displaying road signs in Chinese along the traffic routes to major tourist cities, providing Chinese guidance services in major transportation hubs, and equipping major scenic spots with Chinese self-help explanation devices. DMOs should establish formal collaboration mechanisms with local transportation departments and tourism boards to develop a detailed plan for installing Chinese-language road signs. The goal should be to increase the coverage of Chinese signs on major traffic routes and popular tourist areas in key cities (e.g., Kuala Lumpur, Penang, Melaka) within the next two years. Additionally, during peak tourist seasons (such as Chinese New Year, China’s National Day, and the months of January-February and July-August), bilingual Chinese-English service personnel should be recruited and trained to provide support at major attractions (e.g., the Petronas Towers in Kuala Lumpur, Kek Lok Si Temple in Penang, St. Paul’s Hill in Melaka) and key transportation hubs (e.g., Kuala Lumpur International Airport, Penang International Airport). These personnel should offer 24/7 Chinese-language tours and consultation services. Furthermore, DMOs should develop an official Chinese-language tourism app that provides real-time navigation, attraction information, and emergency assistance features. Additionally, DMOs can provide cultural sensitivity training for staff to improve understanding of the unique expectations and communication styles of Chinese tourists, thereby enhancing the overall visitor experience. DMOs may also collaborate with Chinese travel agencies to ensure that promotional materials, including websites and brochures, are available in Chinese, aligning the destination’s image with the perceptions of Chinese tourists. Staff training in cultural sensitivity can further align communication with Chinese tourists’ expectations, reducing misunderstandings that fuel disappointment or fear, as noted in our findings. Such actions contribute to a positive national tourism image, reduce potential misunderstandings, and enable Chinese tourists to navigate the destination independently and comfortably. To assess the impact of these measures, DMOs could conduct visitor satisfaction surveys and analyze feedback from Chinese tourists regarding their language-related experiences. This would facilitate continuous improvement of language services.
2. To deepen Chinese tourists’ understanding of local religion and culture: Tourism administrative departments should promote local customs, ethnic diversity, and religious cultures indigenous to Malaysia as part of the tourism image to address cultural misunderstandings contributing to image discrepancy. Local travel agencies, tour guides, and scenic spots should also actively promote indigenous cultural image while providing excellent tourist services. Beyond showcasing Malaysia’s multicultural background, DMOs might consider offering specialized cultural immersion experiences—such as guided tours of religious sites and cultural festivals—to provide tourists with more meaningful engagement. DMOs should develop a targeted cultural promotion plan, prioritizing Chinese-language immersive cultural experiences at popular tourist attractions frequented by Chinese visitors (e.g., the UNESCO World Heritage Site of George Town in Penang, the historic city of Melaka, Batu Caves in Kuala Lumpur, and Mount Kinabalu in Sabah). These programs should include guided tours focused on cultural activities, such as religious festivals (e.g., Eid al-Fitr, Deepavali) and traditional village experiences. Additionally, we recommend introducing VR/AR experiences at major religious sites (e.g., the National Mosque in Kuala Lumpur, Cheng Hoon Teng Temple in Melaka, Kuan Yin Temple in Penang) with bilingual Chinese-English content. Furthermore, DMOs should collaborate with local cultural institutions to create and distribute a Chinese-language cultural handbook that covers the origins and significance of Malaysia’s multicultural heritage. These handbooks should be distributed at major entry points and hotels. DMOs could further support cultural understanding by creating multilingual educational content, including videos and interactive exhibits, at key tourist locations to convey the significance of Malaysia’s cultural and religious landmarks. Additionally, implementing immersive technologies, such as virtual reality (VR) or augmented reality (AR), at cultural sites can offer tourists a more engaging and memorable exploration of Malaysia’s heritage. Effective communication of Malaysia’s multicultural identity may also help counteract any negative media coverage, enhancing Chinese tourists’ appreciation for Malaysia’s unique culture. Enhancing cultural recognition among Chinese tourists could strengthen their likelihood of revisiting Malaysia post-pandemic. To monitor the success of these initiatives, DMOs should establish visitor feedback systems to track changes in satisfaction with cultural experiences and identify areas for improvement. DMOs should conduct annual satisfaction surveys targeting Chinese tourists, with survey content including ratings on language services and cultural experiences. Feedback should also be gathered through platforms such as “Weibo” and the “red note” to guide DMOs in adjusting language support and cultural promotion strategies. Additionally, to alleviate disappointment and fear identified as contributing factors, DMOs should establish Chinese-language information centers at major attractions, offering real-time itinerary suggestions to reduce uncertainty and enhance revisit intentions. However, it is important to note that new forms of tourism, such as medical, agricultural, and sports tourism, may not be appealing to Chinese tourists at present, and thus, major efforts should not be devoted to them.

### Limitations and further work

#### Limitation.

First, this study lacks detailed guidance on how the findings can be effectively implemented in the context of destination management and marketing strategies, particularly in enhancing tourist arrivals and reshaping destination image. Secondly, although the sample size does not directly limit the depth of theoretical insights [71], the geographical scope of this study is relatively narrow, which may affect the generalizability of the findings. The Group A respondents were limited to Shanghai and Suzhou, excluding major source cities such as Beijing or Guangzhou. Tourists from northern cities (e.g., Beijing) may be more sensitive to negative news due to cultural preferences or differences in information channels compared to those from southern cities. This could impact the generalizability of the findings in this study. This is a limitation in understanding how tourists from different regions respond to negative news. Third, this study employs the CAGT methodology to extract key factors influencing tourism image from the natural responses of interviewees. Since the themes of the interview data are shaped by participants’ subjective experiences, in-depth exploration of the interplay between economic and political contexts, negative news, and tourism behavior was somewhat limited. Additionally, the short-term nature of negative news effects and the restricted scope of social media responses hindered a comprehensive analysis of its long-term impact. As a result, negative news was not identified as a primary factor in this study. Instead, the research focused on more prominent factors, such as cultural misunderstandings and language barriers, which have been shown to significantly affect visitor experiences. It is worth noting that negative news, when compounded by factors like cultural misunderstandings and economic conditions, may exacerbate its adverse effects. Fourth, this study also lacks a thorough examination of the interaction between negative news and other influential factors, such as economic conditions and political dynamics, which may constrain a holistic understanding of tourists’ perceptions and behaviors. Notably, the interview sample did not include certain groups whose travel or investment decisions are often more sensitive to economic and political contexts, such as middle- and high-income tourists, long-term expatriates, high-end tourism and investment travelers, and tourism industry professionals. These groups tend to consider factors like exchange rates, visa policies, diplomatic relations, and investment environments when making travel or investment decisions. Consequently, the study may not fully capture the unique perspectives of individuals for whom these background factors play a decisive role in tourism choices. Fifth, this study integrates the perspectives of Group A tourists and Group B practitioners, but it does not explicitly explore the potential impact of the weight distribution between these two groups on the theoretical framework. For instance, if practitioners’ professional experiences are overly emphasized, it may underestimate the tourists’ subjective perceptions, and vice versa. This could limit a comprehensive understanding of the causes of image discrepancy. Similarly, our criticism of DMOs’ overemphasis on emerging sectors, such as medical tourism, relies on Group B interviews and promotional materials, such as the promotion of the Malaysia Healthcare program. However, the presentation of specific examples and quantitative data is insufficient.

#### Further work.

First, future research should focus on exploring how the findings of this study can be effectively applied to destination management and marketing strategies, specifically in enhancing tourist arrivals and reshaping the destination image. Secondly, to improve the generalizability of the findings, Future research should expand to a broader geographical scope, incorporating tourists from more Chinese source cities, in order to explore regional differences in tourists’ responses to negative news. Additionally, the weighting of perspectives from Group A and Group B should be optimized to enhance the balance in analysis. Thirdly, future research could consider employing a larger sample size or a mixed-methods approach or mixed-methods approach, combining quantitative data with qualitative interviews, to gain a deeper understanding of the complex interactions between negative news and cultural, economic, and political factors. This would further validate the image discrepancy model proposed in this study and test the generalizability of the CAGT methodology. Furthermore, future studies could benefit from a deeper exploration of how negative news interacts with other factors, such as cultural misunderstandings, economic conditions, and political contexts, to influence tourist perceptions and behaviors. Specifically, understanding how cultural context, economic perceptions, and political environments shape responses to negative news could provide valuable insights for DMOs seeking to refine their image management strategies. Additionally, it is important to consider how cultural misunderstandings may interact with negative media coverage to amplify its effects [20]. Tourists with limited knowledge of Malaysia’s cultural and religious practices may be more inclined to interpret negative news as indicative of broader social or political issues, thereby reinforcing their pre-existing misconceptions [93]. This suggests a potentially complex interplay between media influence and pre-existing cultural misunderstandings, which future research could explore in greater depth [90]. Finally, future research should consider extending the sample of interview subjects to include groups that are more concerned with economic and political contexts, such as high-income tourists, expatriates, high-end leisure or investment tourists. These groups are more attuned to factors such as travel costs, consumption levels, economic conditions, political stability, and policy changes. Compared to general tourists, these groups are more directly influenced by exchange rates, visa policies, diplomatic relations, and investment environments, thus offering unique insights into how these factors affect tourism choices. Finally, future research should further validate the impact of DMOs’ strategies on image discrepancy by incorporating more comprehensive promotional data and quantitative metrics (e.g., budget allocation or visitor feedback statistics).

## Conclusion

This study, through in-depth interviews and the CAGT methodology, explores whether sudden negative news events significantly impact Chinese tourists’ travel decisions and the recovery of tourist arrivals to Malaysia. It also analyzes the specific factors contributing to the discrepancy between Chinese tourists’ perceived image and the image projected by DMOs. The study finds that the impact of negative news and the resulting negative emotions is relatively small. Cultural differences and language barriers limit Chinese tourists’ understanding and appreciation of Malaysia’s multiculturalism, leading to a disconnection between the perceived and projected images. Secondary factors include the low efficiency of DMOs in promoting a multi-ethnic image and the ineffective projection of emerging sectors, such as medical tourism.

From a theoretical perspective, this study expands the application of the image discrepancy theory in the context of cross-cultural tourism. The CAGT methodology, through systematic analysis of qualitative data, reveals the deeper causes of image discrepancy. Particularly in situations with limited data, it offers a unique advantage in studying the impact of tourists’ emotional responses to sudden events on tourist arrivals.

From a practical standpoint, this research provides clear strategic recommendations for DMOs. First, DMOs should prioritize addressing language barriers by implementing measures such as displaying Chinese road signs in major tourist cities, offering Chinese-language tour guides, and providing Chinese-language self-guided audio devices to enhance the experience for Chinese tourists. Second, DMOs should strengthen the promotion of local and religious culture by offering cultural immersion programs and multilingual educational content to increase tourists’ awareness of Malaysia’s multicultural heritage. Additionally, it is recommended that DMOs avoid over-investing in emerging sectors, such as medical tourism, which have limited appeal, and instead focus on showcasing tourism images that highlight the region’s ethnic characteristics. This will help reduce image discrepancy and enhance tourist arrivals. We hope that these findings will help tourism destinations, including Malaysia, better attract Chinese tourists and promote the sustainable development of the tourism industry.

## Supporting information

S1 File Open coding of perceived tourism image. (PDF)

S2 File Selective coding of perceived tourism image. (PDF)

S3 File Theoretical coding of perceived tourism image. (PDF)

S4 File Open coding of projected tourism image. (PDF)

S5 File Selective coding of projected tourism image. (PDF)

S6 File Theoretical coding of projected tourism image. (PDF)
