# Supplementary material for: Navigating the image discrepancy: A grounded theory approach to understanding Malaysia’s image among Chinese tourists
Source: PLoS One. 2025 May 27;20(5):e0324148. doi: 10.1371/journal.pone.0324148 (PMC12111393; doi:10.1371/journal.pone.0324148)
Supplement: S1 File — (PDF) [file pone.0324148.s001.pdf]

Open Coding of Perceived Tourism Image

- **an image that is not clearly perceived**
  - The Influence of Language on Image Perception
    - Accessibility of Chinese Language Materials
    - Customs declaration forms are provided in Chinese upon entry, simplifying administrative processes.
    - Tourists can frequently use Mandarin and Chinese characters during their journey
      - Most tourist attractions feature Chinese-language signage for easy navigation
      - Communication with the local Chinese community is seamless and fluent.
  - Ambiguity in Understanding Ethnic, Religious, and Local Cultural Images
    - Vague Impressions of Malaysia's Multi-Ethnic Identity
      - Islamic Cultural Image
        - Interest in the dietary habits of local Malay
          - Tour guides did not arrange visits to Halal restaurants.
          - While aware that using the left hand for eating is inappropriate, there is a desire to experience authentic local dining options instead of sticking to Chinese restaurants.
    - Limited Understanding of Religious Culture
      - Insufficient Knowledge of Islamic Culture
        - Uncertainty about whether it is appropriate to travel to Malaysia during Ramadan.
        - Lack of clarity on how non-Muslims should behave during Muslim prayers.
        - Questions about proper etiquette when visiting mosques.
      - Respecting Muslim Customs
        - Tour guides did not provide instructions on how to greet Muslims
        - Tour guides advised against shaking hands with Malay women but did not explain alternatives for respectful greetings
        - Uncertainty about how to properly greet local Muslim women.
        - Questions about the correct way to address Malaysian Muslim friends by their names.
    - Limited Understanding of Local Tourism Culture
      - Interest in experiencing the daily lives of local residents in rural areas and non-tourist cities
        - Tourist destinations are limited to famous attractions and major cities.
        - Non-tourist cities leave no lasting impression.
        - Group tours rarely provide opportunities to visit rural areas.
      - Fascination with the tourism appeal of other ethnicities but lack of clear understanding
        - Islamic and Hindu cultural elements are absent from tourist itineraries
          - Hindu temples, despite their stunning architecture, are not included in tour routes
            - Hindu temple architecture is beautiful, but the sites were not visited.
            - Some temples were observed from the outside but not visited as part of the itinerary.
          - Respect and curiosity for Islam:
            - Difficulty accessing mosques:
              - Interest in visiting mosques, but such visits are not included in travel packages.
              - Mosques appear beautiful from the outside, but tourists are unsure how to enter or visit them respectfully.
              - Tour guides lack knowledge about how to arrange mosque visits.
            - Islamic villages with unique characteristics are often excluded from tours.
        - Lack of Religious Diversity in Itineraries.
- **The cultural image of multi-ethnic integration and development.**
  - Ethnic minorities have integrated into local life and formed new cultural identities.
    - Integration of Chinese Local Culture into Malaysian Culture
      - Distinctive Features in Religious and Festive Traditions:
        - Chinese festivals are celebrated with unique Malaysian adaptations:
          - The Lantern Festival's "Throwing Oranges and Bananas" (Pao Gan Jie Jiao) is an exclusive Malaysian Lantern Festival tradition.
      - Nyonya Culture as a Microcosm of Integration
        - Nyonya cuisine represents Malaysian-style Chinese dishes.
        - Nyonya culture exemplifies the localization and continuation of Chinese cultural heritage in Malaysia.
        - Nyonya attire reflects a uniquely Malaysian interpretation of traditional Chinese clothing.
    - Most Mamak Stalls are operated by Indian Malaysians, but their patrons come from all ethnic backgrounds.
      - At a Mamak Stall, you can observe people from various ethnicities dining together.
    - Masjid Jamek: embodies a fusion of Indian and Islamic architectural styles.
  - Ethnic harmony and coexistence
    - Mutual respect and love exist among the diverse ethnic groups, fostering harmonious coexistence.
      - On the streets, it is common to see young people from different ethnicities engaging in friendly interactions.
      - People respect each other's customs and festivals, often celebrating together:
        - Deepavali, although an Indian festival, is celebrated nationwide.
        - During Hari Raya (Eid al-Fitr), Chinese and Indian communities also participate in the celebrations.
        - During Chinese New Year, Malays and Indians join in the festivities with us.
    - Malaysia is a multi-ethnic country
      - Minority groups receive full respect.
        - Hindu temples are well-preserved.
        - Chinese people have made significant contributions throughout Malaysia's history.
      - Chinese temples, Hindu temples, and mosques are located on the same street.
      - the image of Islam serving as one of the key representations of its local identity
        - An Ideal Destination for Muslim Travelers:
          - Comfortable living and dining conditions for Muslims
            - Halal food labeling is clear and easily identifiable.
          - The dietary habits of Malays align with those of Chinese Muslims.
          - Hotel rooms are equipped with "kiblat" arrows to indicate the direction for prayer.
        - A Healthy, Positive, and Confident Social Image
          - Islam promotes healthy dietary practices
            - Dietary habits align with modern scientific nutritional principles
              - Halal food is minimally processed
              - The proportions of protein, carbohydrates, and fats are balanced.
              - Halal cuisine emphasizes natural flavors.
              - There is minimal use of artificial additives.
          - Emphasis on reducing desires and striving for self-improvement.
        - Mosque designs reflect the tranquility and grandeur of Islam
          - Color schemes in mosques evoke a sense of inner peace.
          - Architectural styles are dignified and elegant.
- **basic functional image**
  - pre-condition
    - The level of tourism safety in Malaysia falls between that of Thailand and Singapore, with major cities in West Malaysia being relatively safer.
      - Malaysia is considered safer than Thailand.
      - Psychologically, West Malaysia feels slightly more secure.
      - Tourism safety in major cities is generally well-assured.
      - Malaysia's overall sense of safety does not match that of Singapore, which is a single city-state.
    - The infrastructure in major cities in West Malaysia is particularly reliable.
      - There is significant variation in the quality of infrastructure accessible to tourists, with cities, especially in West Malaysia, being better equipped.
      - Infrastructure in major cities in West Malaysia is of a high standard.
        - Urban infrastructure is well-developed and comprehensive.
          - Major cities boast world-class infrastructure.
        - Infrastructure in West Malaysia is comparable to that of China, while East Malaysia lags behind in this regard.
      - The hygiene environment in West Malaysia better meets tourists' expectations.
        - Overall, the hygiene standards in tourist areas are acceptable, but East Malaysia falls behind West Malaysia in this aspect.
  - favorable conditions
    - Chinese electronic payment methods and UnionPay cards are widely accepted for shopping in Malaysia.
      - Purchases over 50 MYR in duty-free shops can be made using these payment options.
      - Alipay is accepted in major shopping malls.
      - UnionPay credit cards are highly convenient:
        - Most malls allow transactions with UnionPay credit cards.
    - A cost-effective travel destination
      - A low-cost destination offering great value for money
      - A clean and worthwhile destination.
      - Affordable prices for a wide variety of cuisines.
    - The residents are kind, hospitable, and straightforward in their demeanor, creating a friendly atmosphere for tourists.
      - Easy communication
        - Simple and hospitable local culture with distinctive characteristics:
          - Locals are humble and polite.
        - High language proficiency among the Chinese community:
          - The presence of Chinese locals feels familiar and welcoming:
            - Seeing many Chinese people in Malaysia creates a sense of closeness.
            - The customs of Malaysian Chinese are nearly identical to those of Chinese tourists.
            - Hotels feel friendly, often employing Chinese-speaking staff.
          - Many Chinese locals are multilingual, allowing for communication in Mandarin.
          - Many small vendors and tourism workers speak English:
            - Boat operators at marine parks and other businesspeople interact with visitors using simple English.
      - Positive social atmosphere:
        - Malaysian businesses are honest:
          - No instances of consumer fraud
        - Greater integration among young people of different ethnic groups.
        - Advertisements often feature Malays, Chinese, and Indians together, reflecting cultural harmony.
- **an anticipated service image.**
  - Accommodation is satisfactory.
    - Staying in homestays allowed me to experience Malaysian culture.
    - Hotels are clean and hygienic.
  - An ideal destination for Muslim travelers
    - Comfortable living and dining conditions for Muslims:
      - Halal food labeling is clear and easily identifiable.
    - The dietary habits of Malays align with those of Chinese Muslims.
    - Hotel rooms are equipped with "kiblat" arrows to indicate the direction for prayer.
  - Tourist Souvenirs:
    - Pewter souvenirs left a strong impression on me
      - Malaysia's pewter wine flasks are particularly beautiful.
    - Exquisite handicrafts.
  - Colorful and Diverse Food Culture
    - Traditional Cuisine Blended with Multicultural Characteristics
      - Malaysian cuisine integrates the cultures of Chinese, Indian, and Malay communities.
      - Chinese food is exceptionally delicious.
      - I really enjoy traditional foods.
    - Southeast Asian Flair in Culinary Delights
      - Malaysian cuisine is characterized by spicy, sour, and sweet flavors.
      - Spicy and appetizing dishes.
    - Halal Food is Very Healthy
    - White Coffee is a Unique Coffee Specialty of Malaysia
- **A rich and colorful tourist aesthetic image**
  - A Blend of Ancient and Modern Architectural Styles
    - Ancient Architecture Reflecting Historical Heritage
      - Buildings from the colonial era are worth visiting:
        - Represent Malaysia's history of independence and development:
          - Sultan Abdul Samad Building in Kuala Lumpur
          - Historical facades near Dataran Merdeka (Independence Square) in Kuala Lumpur
        - Colonial architecture in Penang leaves a lasting impression
        - Fort Cornwallis is a must-visit:
          - A beautiful example of British architecture
      - Well-preserved structures of various religious forms
        - Numerous well-maintained mosques
        - St. Mary's Cathedral
        - Chinese traditional architecture is also prominent
          - Sin Sze Si Ya Temple (仙四师爷庙) in Kuala Lumpur
          - Snake Temple in Penang is unique and unlike anything in China:
          - Snake Temple is a Chinese-style building
          - Buddhist temples have differences from those in China
    - Modern Architecture Famous Worldwide
      - Modern Architecture in Kuala Lumpur
        - Kuala Lumpur Tower
        - Petronas Twin Towers
      - Iconic Landmarks That Leave a Lasting Impression
        - The Petronas Twin Towers are highly modern and innovative.
  - High Level of Modern Technology and Culture
    - Putrajaya: A Modern High-Tech City
      - Famous for its hot air balloons.
    - Multimedia Super Corridor
      - A hub of advanced technology.
  - Rich Local Cultural Tourism Elements
    - Indigenous Tourism: Mah Meri Ethnic Group
      - Mah Meri Cultural Village
      - Unique indigenous handicrafts (weavers, woodcarvers, and dancers)
    - Distinctive Festivals
      - Impressive festivals in Penang
      - Unique celebrations in Sarawak
    - Kampung Village Culture
      - Deeply impressed by the charm of Kampung rural culture.
  - Natural and heritage tourism image
    - The natural tourism resources are diverse and vibrant
      - The Unique Natural Environment of Cameron Highlands
        - Bertam River
        - Cactus Valley
      - Tropical Scenery
        - Impressive Tropical Rainforests
        - Beautiful Islands
          - The seas in East Malaysia are even more stunning.
            - Attractive Coastal Beaches
          - Tioman Island,
            - Clear seawater
            - Beautiful beaches
          - Langkawi
            - Langkawi is breathtaking
      - Unique Ecotourism in Borneo's Taman Negara
      - Johor's Mangrove Wetland Landscapes
        - Pulau Kukup State Park
        - Ramsar Wetlands in Johor
        - Sungai Pulai Forest Reserve
        - Tanjung Piai State Park
    - Aesthetic cultural image of world heritage
      - Cultural Heritage: A Glimpse into Malaysia's Historical Development
        - Tourist Destinations Showcasing the Wisdom of Early Settlers
          - Lenggong Valley (World Heritage Site)
            - Ancient human archaeological remains.
          - Malacca (World Heritage Site): The historic city is a must-visit.
        - George Town (World Heritage Site)
          - The famous street murals of George Town.
      - Natural Heritage: Tropical Scenery That Leaves Visitors in Awe
        - Impressive Landforms at Gunung Mulu National Park
        - Kinabalu Park (World Heritage Site): Stunning tropical landscapes.
        - The natural scenery of Kinabalu Park is breathtaking.
        - The natural beauty of Gunung Mulu National Park is captivating.
        - Gunung Mulu National Park (World Heritage Site)

Presented with xmind
