# Supplementary figures and images for: Navigating the image discrepancy: A grounded theory approach to understanding Malaysia’s image among Chinese tourists
Source: PLoS One. 2025 May 27;20(5):e0324148. doi: 10.1371/journal.pone.0324148 (PMC12111393; doi:10.1371/journal.pone.0324148)

Selective coding of perceived images

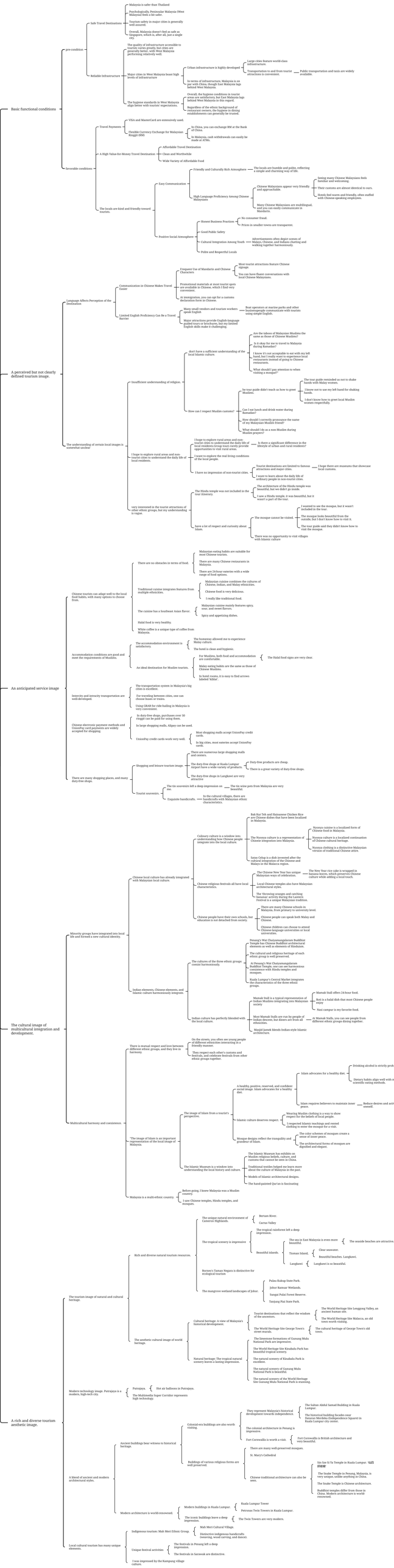

Supplement: S2 File — (PDF) [file pone.0324148.s002.pdf]

Theoretical Coding of Perceived Images

tourists focus

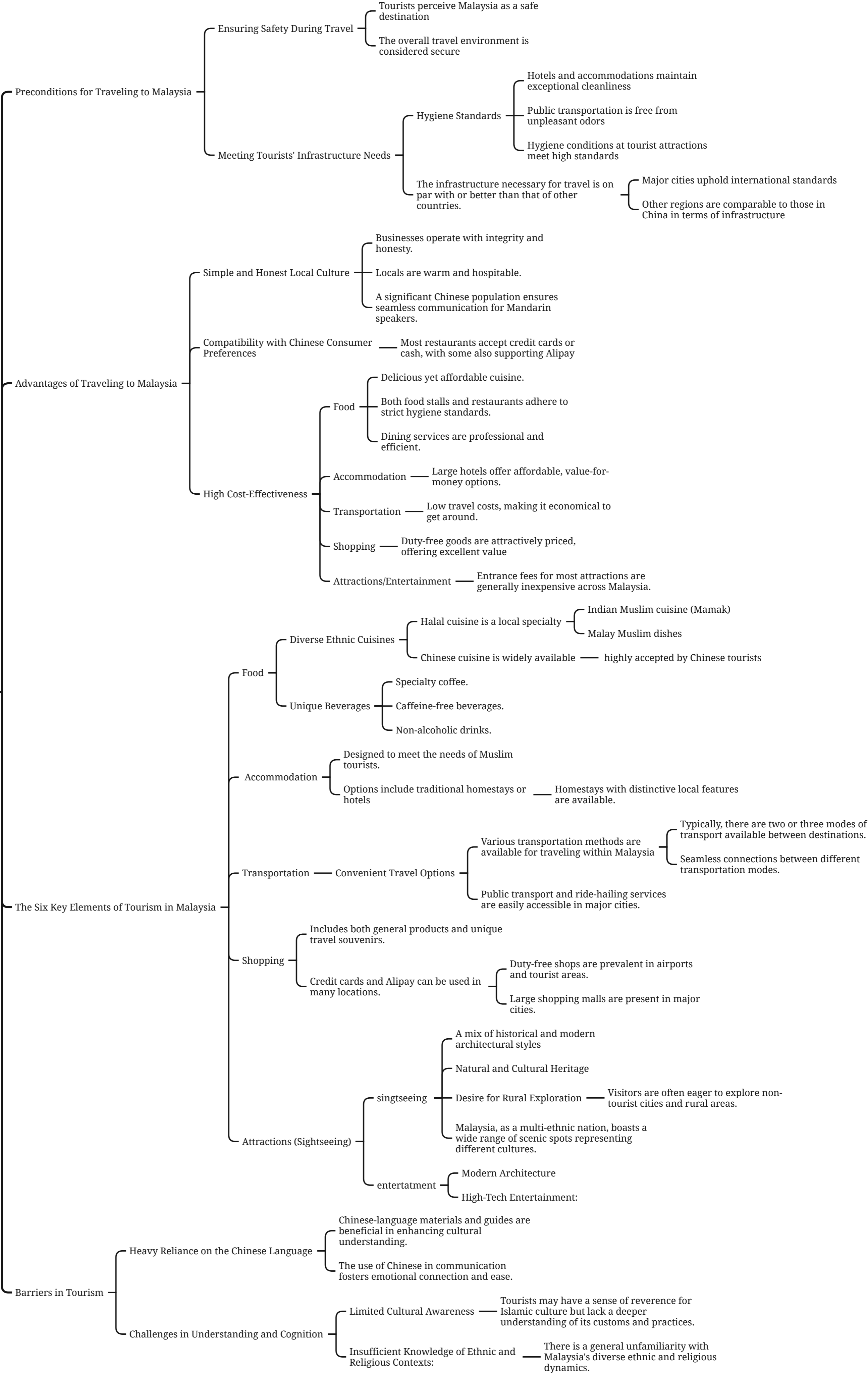

Supplement: S3 File — (PDF) [file pone.0324148.s003.pdf]

Open coding of projected images

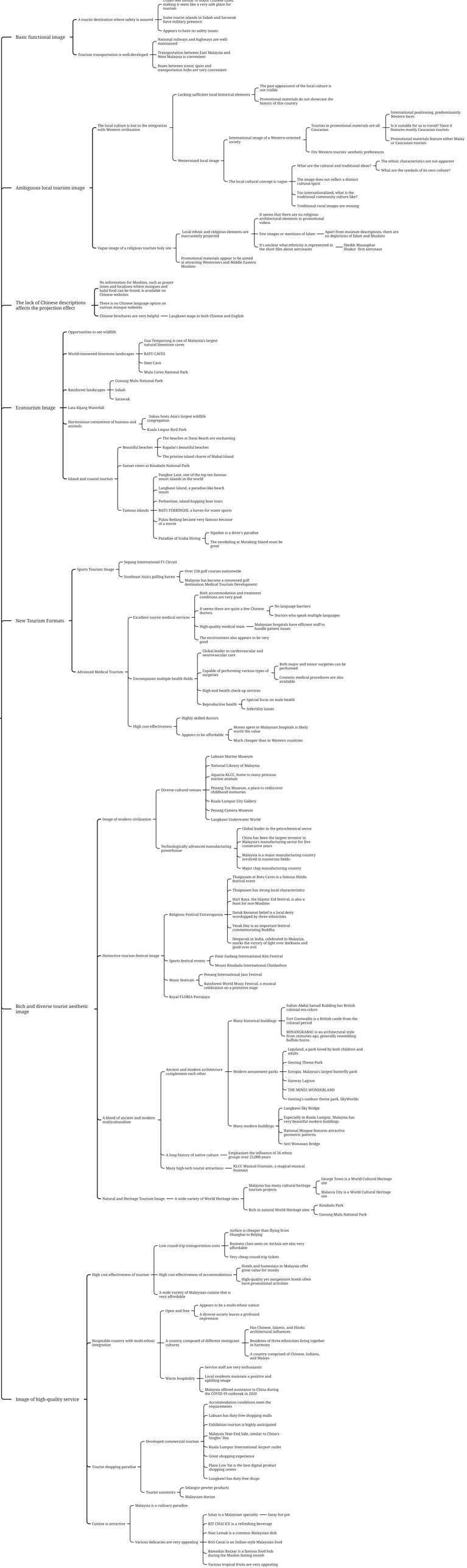

Supplement: S4 File — (PDF) [file pone.0324148.s004.pdf]

selective coding of projected images

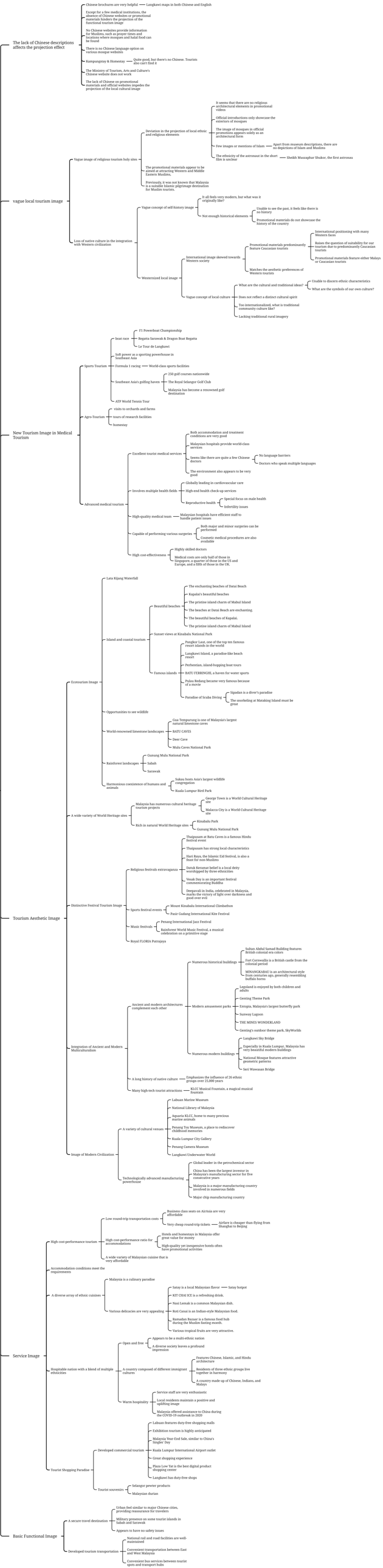

Supplement: S5 File — (PDF) [file pone.0324148.s005.pdf]

theoretical coding  
of projected images

host focus

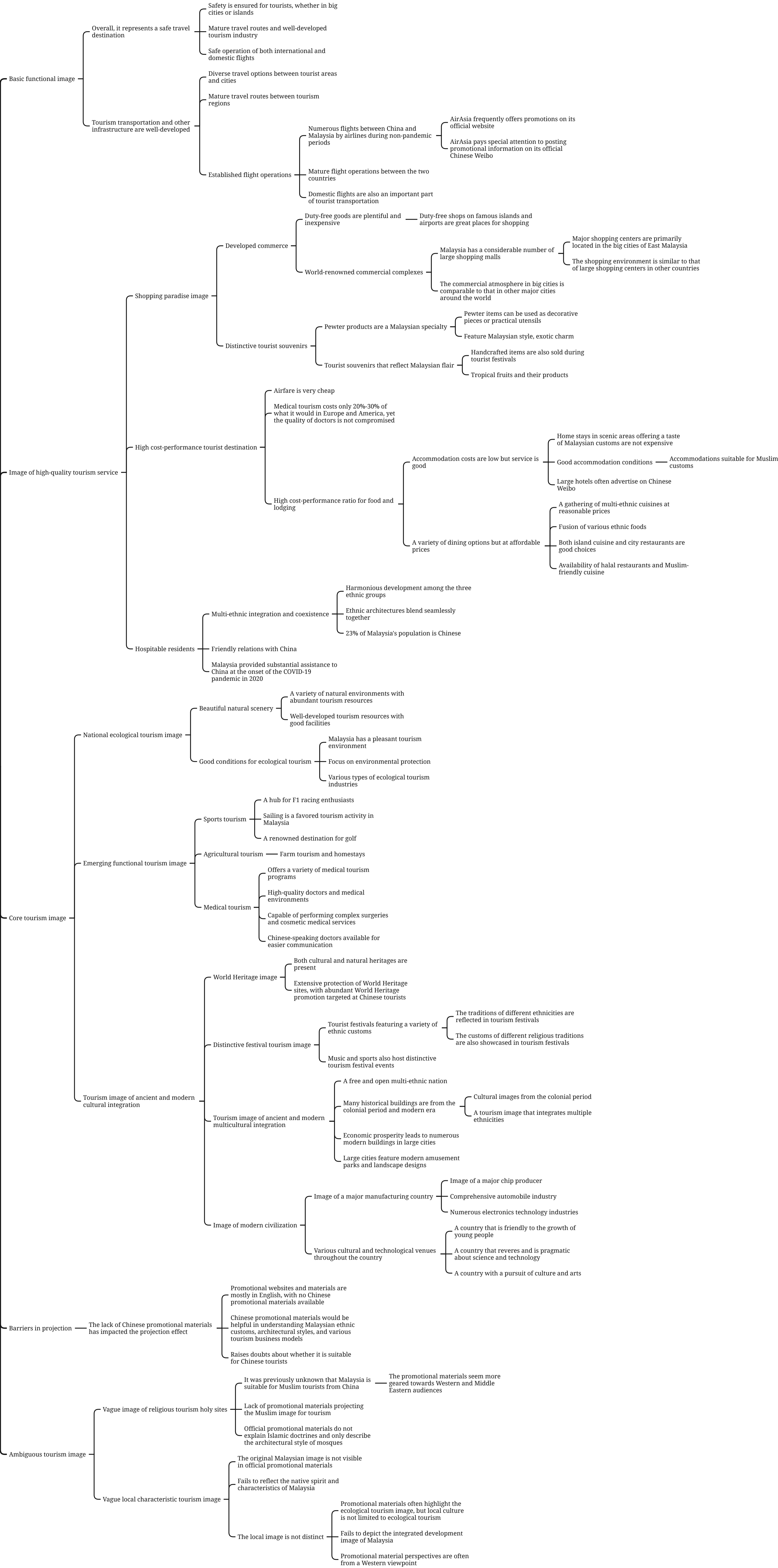

Supplement: S6 File — (PDF) [file pone.0324148.s006.pdf]
